# An all-in-one nanoprinting approach for the synthesis of a nanofilm library for unclonable anti-counterfeiting applications

**DOI:** 10.1038/s41565-023-01405-3

**Published:** 2023-06-05

**Authors:** Junfang Zhang, Yuxin Liu, Christian Njel, Sebastian Ronneberger, Nadezda V. Tarakina, Felix F. Loeffler

**Affiliations:** 1 Max Planck Institute of Colloids and Interfaces, Potsdam, Germany; 2 Department of Chemistry and Biochemistry, Freie Universität Berlin, Berlin, Germany; 3 Institute for Applied Materials (IAM) and Karlsruhe Nano Micro Facility (KNMFi), Karlsruhe Institute of Technology (KIT), Eggenstein-Leopoldshafen, Germany; 4 Institute of Physics and Astronomy, University of Potsdam, Potsdam, Germany

**Keywords:** Synthesis and processing, Surface patterning, Chemical engineering, Quantum dots

## Abstract

In addition to causing trillion-dollar economic losses every year, counterfeiting threatens human health, social equity and national security. Current materials for anti-counterfeiting labelling typically contain toxic inorganic quantum dots and the techniques to produce unclonable patterns require tedious fabrication or complex readout methods. Here we present a nanoprinting-assisted flash synthesis approach that generates fluorescent nanofilms with physical unclonable function micropatterns in milliseconds. This all-in-one approach yields quenching-resistant carbon dots in solid films, directly from simple monosaccharides. Moreover, we establish a nanofilm library comprising 1,920 experiments, offering conditions for various optical properties and microstructures. We produce 100 individual physical unclonable function patterns exhibiting near-ideal bit uniformity (0.492 ± 0.018), high uniqueness (0.498 ± 0.021) and excellent reliability (>93%). These unclonable patterns can be quickly and independently read out by fluorescence and topography scanning, greatly improving their security. An open-source deep-learning model guarantees precise authentication, even if patterns are challenged with different resolutions or devices.

## Abstract (web summary)

A laser printing approach generates physical unclonable fluorescent patterns, made from simple sugar. These environmentally friendly and ultraviolet-stable materials can be applied as anti-counterfeiting labels.

## Main

Counterfeiting is a serious global problem that causes trillion-dollar losses to industry, and these losses are increasing annually^1^. Furthermore, counterfeiting in pharmaceuticals^2^, certificates^3^ and electronics^4^ directly threatens human health, social equity and national security. One highly effective solution is anti-counterfeiting labelling, especially easily verifiable optical security devices, such as fluorescent holograms^5^. A considerable number of fluorescent materials have been explored for this purpose, including semiconductor quantum dots^6^, organic dyes^7^ and carbon dots (CDs)^8^. Among these materials, CDs stand out in particular because of their stability, low toxicity, widely available precursors and bio-/ecofriendly preparation^9^. However, most reported CDs only fluoresce in solution, and suffer from *π*–*π* stacking and quenching in the solid state^10^. Doping CDs into matrices (for example, polymers, salts or starch)^11,12^ or synthesizing self-dispersive CDs (for example, by steric hindrance, electrostatic repulsion or hydrogen bonding)^8,13–15^ are state-of-the-art strategies to achieve solid-state fluorescence (SSF). CD monomers are blocked from direct interaction to avoid aggregation-induced quenching. Nevertheless, most obtained CDs show a substantial spectral shift with competitive non-radiative decay or obvious concentration-dependent luminescence characteristics^16^. This results in decreased quantum efficiency or additional optimization steps for desired emission colours in the solid state.

Recently, reversible responsiveness has been introduced to improve the security level of optical anti-counterfeiting labels by implementing multiple operation modes^17–22^. Degradation side-products are prone to form after repeated mode switching^7^. Furthermore, after the fluorescent compound is disclosed, most of these techniques, however costly and sophisticated, can be copied within 18 months^3,23^. Therefore, encryption by physical unclonable functions (PUFs), such as identifiable macropatterns with unpredictable microstructure^24,25^, provides a facile solution to make optical security devices easy to verify but challenging to forge^26,27^. PUFs are fabricated by stochastic processes to guarantee uniquely random patterns^28^. For anti-counterfeiting labelling, PUFs are typically generated by rough surfaces^6,29,30^ or discrete nanoparticle arrays^31,32^ within a predefined area. Although they show high security levels, PUF patterns always require either tedious fabrication or complex readout methods, which hamper their practical application.

To overcome the challenges in SSF materials and PUF patterns, here we propose an all-in-one nanoprinting approach to in situ generate CD nanofilms with multichannel unclonable microstructures. Polymer CD (PCD) patterns are flash synthesized from and stabilized by monosaccharides within milliseconds. The whole process is environmentally friendly (fluorophores are produced from sugar-based material, without the need for toxic precursors or organic solvents), highly efficient and easily tunable, allowing the establishment of an SSF film library with 1,920 experimental datasets. The library comprised both chemical environment and physical printing parameters, providing multiple choices for optical properties and microstructures. Due to the intrinsic randomness, these microstructures may serve as PUF patterns, which can be read out by two independent and fast methods: fluorescence (FL) scanning and white-light interferometry (WLI). Both readouts exhibit sufficient encoding capacity, near-ideal bit uniformity, uniqueness and reliability. The combination of fluorescence and topography microstructures greatly improves the security level of the PUF patterns, validated in 100 individual experiments, making them immune to general attacks such as nanomoulding or nanolithography. Finally, an open-source deep-learning model (LoFTR, Detector-Free Local Feature Matching with Transformers) was adapted and introduced for authentication, which allows precise identification even when end-users scan the PUF patterns with different resolutions or devices.

## Nanoprinting-assisted flash synthesis for in situ SSF

**Fig. 1:**
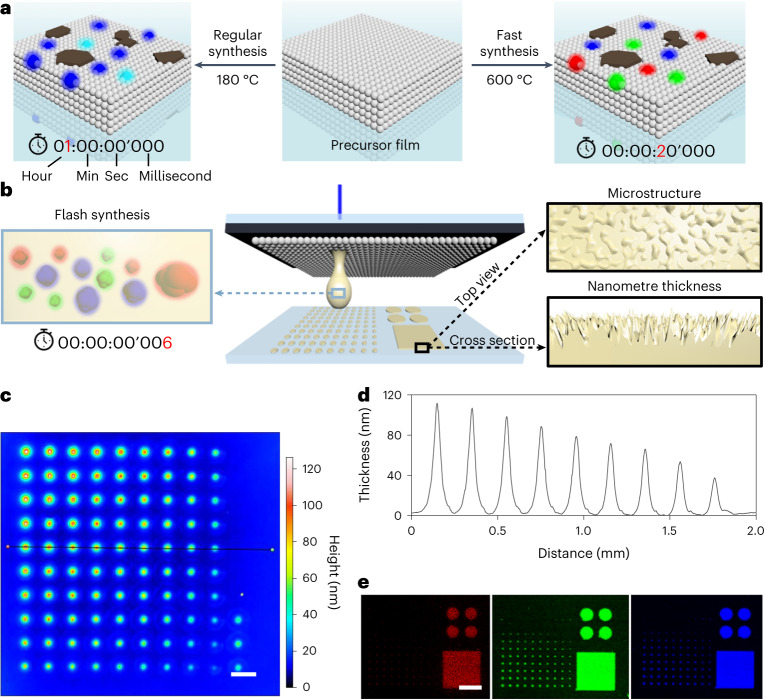
Principle of the nanoFlash synthesis approach and characterization of transferred patterns. **a**, Solvent-free synthesis of fluorescent carbon dots from a glucosamine film in an air oven. **b**, Fluorescent patterns with microstructures were generated by the laser-driven transfer of precursors. **c**,**d**, Height map (**c**) and profile (**d**) of a transferred spot gradient show material height in nanoscale. Scale bar, 200 µm. **e**, Fluorescence scanning using 635 nm (left), 532 nm (middle) and 488 nm (right) excitation channels. Scale bar, 600 µm. For the square area, the bitmap mode was used with 105 mW laser power and 100 mm s^−1^ scanning speed. The gradient began at the right bottom corner (12 mW laser power, 1 ms). The laser power increased by 13 mW per line and the irradiation time increased by 1 ms per row.

Unlike traditional strategies, in which CDs are synthesized and purified in a liquid phase, and then optimized for SSF, we aimed to synthesize CDs directly in the solid phase by a solvent-free approach. We prepared films from the precursor d-(+)-glucosamine hydrochloride on glass slides and heated them in an air oven (Fig. 1a and Supplementary Fig. 1). Notable fluorescence occurred in the water solution of the annealed films and higher annealing temperatures caused a redshift of the fluorescence (Supplementary Fig. 2). NMR spectroscopy (Supplementary Fig. 3) and liquid chromatography–mass spectrometry (Supplementary Fig. 4) indicated that this solvent-free synthesis might shared the same mechanism with the reported liquid-phase synthesis from the same precursors^33^. However, direct SSF of the annealed films was not observed. Although shorter annealing times were applied for higher temperatures, large carbon flakes from overcarbonization appeared on the films (Supplementary Fig. 1a), which could block the excitation and reabsorb emitted light. Therefore, flash synthesis^34,35^ within a subsecond time scale is necessary to in situ generate fluorescent CDs in the solid phase.

Our group focuses on laser-based nanoprinting technologies, which can precisely heat a confined position in milliseconds^36^. After introducing a nanolayer laser absorber, we greatly improved the resolution of the nanoprinting technology^37^. By selecting film-forming precursors (for example, monosaccharides), we could now omit the supporting polymer matrix—essential for printing in our previous works—during ink preparation. Based on this, we developed a nanoprinting-assisted flash (nanoFlash) synthesis approach (Supplementary Fig. 5), achieving in situ SSF during the ultrafast printing of micro-/nanopatterns (Fig. 1b). Specifically, the monosaccharide solution was spin-coated onto a glass slide with a laser-absorber layer. During the laser-printing process, the absorber layer converted the laser pulses into heat, achieving a temperature above 500 °C^38^. The precursor was melted and transferred onto another substrate. The thickness of the transferred patterns was tunable in the nanoscale (Fig. 1c,d). The ultrafast annealing process avoided overheating and the formation of large carbon flakes on the printed film. Without any post-treatment, SSF was observed in the directly transferred pattern (Fig. 1e). The fluorescence signal from the red (635 nm), green (532 nm) and blue (488 nm) channels (RGB channels) showed a clear response to the change of laser parameters. Moreover, for the printing of areas, the nanoFlash synthesis approach offers extremely fast scanning speeds of up to hundreds of mm s^−1^ (Supplementary Video 1). Scanning electron microscopy revealed that distinct microstructures appeared on the transferred areas, and their structures could be tuned by the laser intensity (Supplementary Fig. 6a,b). Notably, we placed the obtained patterns under ultraviolet light (285 nm) for up to 15 h, without observing fluorescence quenching (Supplementary Fig. 6c–e). A thin protection layer may be introduced on top of the patterns by spin coating, making them waterproof (Supplementary Fig. 7).

## Nanofilm characterization and high-throughput library

**Fig. 2:**
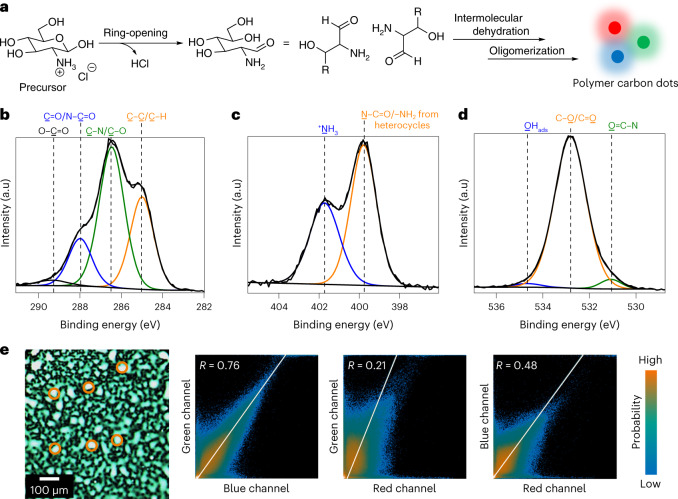
Chemical and optical analysis of the nanofilms obtained by the nanoFlash method. **a**, Proposed reaction process of PCDs. **b**–**d**, High-resolution C 1*s* (**b**), N 1*s* (**c**) and O 1*s* (**d**) XPS binding energy spectra of the nanofilm. **e**, Merged fluorescent pattern of the nanofilm. The orange circles highlight examples of strong co-localization of all three fluorescence channels (red + green + blue, adding up to white). Co-localization analysis of different fluorescence channels by Pearson correlation (threshold regression: bisection) shows especially low correlation of the red channel with the other two. Fluorescence acquisition: red channel: excitation, 635 nm; emission, 659–701 nm; green channel: excitation, 532 nm; emission, 597.5–612.5 nm; blue channel: excitation, 488 nm, emission, 517.5–522.5 nm. Source data

The obtained thin films from the nanoFlash approach were in situ analysed by X-ray photoelectron (XPS) spectroscopy (Fig. 2b–d and Supplementary Tables 1 and 2). In comparison to the precursor film, the carbon/oxygen ratio of the nanoFlash film almost doubled, suggesting hydroxyl group elimination. The C 1*s* spectra (Fig. 2b and Supplementary Fig. 8) show an increase in the peak located at 285 eV, indicating newly formed C–C bonds. In the O 1*s* spectra (Fig. 2d and Supplementary Fig. 8), the decrease of the peak at 532.8 eV gives evidence for the dehydration of the precursors. Meanwhile, new peaks in the ^1^H NMR spectra at 8.4 and 8.6 ppm (Supplementary Fig. 3) prove the formation of heterocyclic structures, which might overlap with the peak at 399.7 eV in the N 1*s* spectrum (Fig. 2c). Another peak at 401.5 eV, mainly assigned to ^+^NH_3_ (Cl^−^), suggests that precursor is still present in the nanoFlash films. Therefore, during the nanoFlash process, parts of the precursor probably undergo ring-opening, elimination of HCl and oligomerization by intermolecular dehydration to form carbon dots (Fig. 2a and Supplementary Fig. 7d–e). Transmission electron microscopy, X-ray powder diffraction and atomic force microscopy (Supplementary Fig. 9) further revealed an amorphous structure of the materials obtained by the nanoFlash approach with particle sizes around 10 nm (for time-resolved photoluminescence spectroscopy and fluorescence anisotropy analysis, see Supplementary Information, section 5). These properties fall within the concept of ‘polymer carbon dots’^13,39,40^, a recently emerging class of carbon dots, which show relatively large particles in contrast to traditional carbon dots (<100 nm versus <10 nm) and high chemical inertness. However, from the in situ TEM measurements, where the fluorescent nanopattern was directly generated on the TEM grid (Supplementary Fig. 10), no carbon core could be observed. Therefore, the PCDs formed in our process are more probably polymeric fluorescent molecules. When dissolved in water and dried on another surface, they aggregated into nanoparticles.

Furthermore, fluorescence patterns of the films were recorded in the RGB channels and merged (Fig. 2f). Two distinct areas, white particles (several highlighted with orange circles) and green wrinkles, can be observed. A co-localization analysis^41^ of the fluorescence channels shows that the green and red channels have a low correlation (*R* = 0.21) compared with the other groups (green–blue, *R* = 0.76; blue–red, *R* = 0.48). Therefore, the red-emitting and green-emitting PCDs show different microstructural distributions in the printed nanofilms. Thermodynamic simulations of the nanoFlash process (Supplementary Fig. 11) ascribe this variation to sluggish heat diffusion and large temperature differences within the precursor layer. Furthermore, the three-dimensional (3D) fluorescence spectra (Supplementary Fig. 12) revealed an excitation-dependent behaviour of the nanofilms, similar to the case of matrix-dispersed SSF^15^. Thus, the PCDs are probably dispersed in the precursor matrix, which strongly reduces Förster resonance energy transfer to avoid aggregation-induced fluorescence quenching in the solid state.

**Fig. 3:**
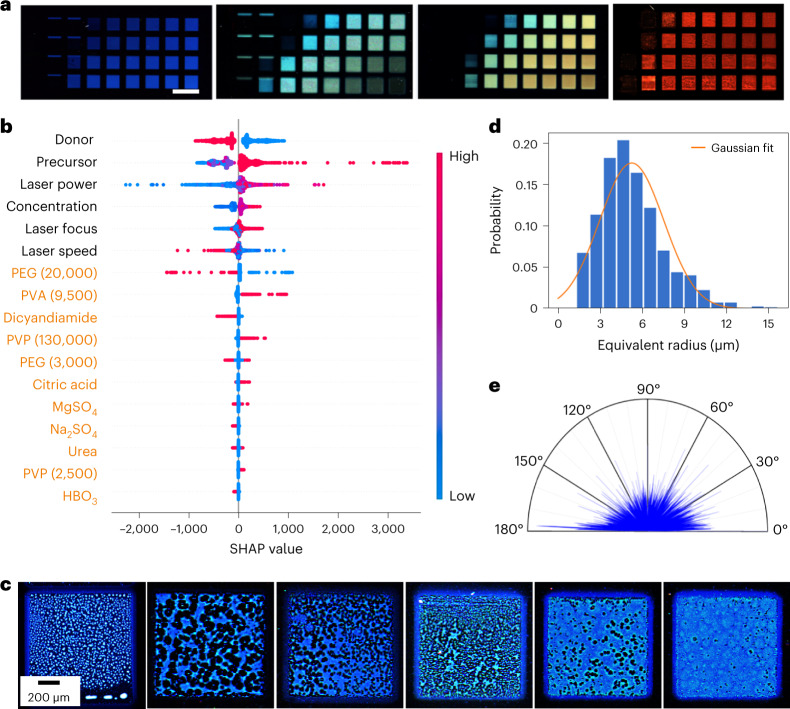
Fluorescent film library achieved by nanoFlash synthesis. **a**, Selected nanofilms from the library (see Supplementary Information Fig. 15 for the whole library) generated with individual synthesis parameters (Supplementary Table 3). Scale bar, 2 mm. **b**, SHAP summary plot for the XGB model, identifying the impact of synthesis parameters on red fluorescence intensity. Value of the feature represented from low (blue) to high (red). Donor: low, Fe_2_O_3_; high, CuO. Precursor: low, *N*-acetylglucosamine; medium low, glucose; medium, galactose; medium high, glucosamine; high, toluenesulfonic acid. Laser power: eight values from low (31 mW) to high (129 mW) (Supplementary Fig. 16). Concentration: low, 50 mg ml^−1^; low medium, 100 mg ml^−1^; high medium, 200 mg ml^−1^; high, 300 mg ml^−1^. Laser focus: low, 4.62 mm; high, 4.32 mm. Laser speed: low, 30 mm s^−1^; low medium, 50 mm s^−1^; high medium, 100 mm s^−1^; high, 150 mm s^−1^. Additives (orange): low, no additive; high, with additive. PVP, polyvinylpyrrolidone. **c**, Films exhibiting different microstructures but similar fluorescence colour. **d**,**e**, Equivalent radii of the particle-like droplets (**d**) (Supplementary Fig. 27a) and the angular orientation distribution of the stripe-like droplets (**e**) (Supplementary Fig. 27b). A 0° angle corresponds to the horizontal direction in the image; 90° corresponds to the vertical direction. Source data

The fluorescence spectra of the films obtained with different parameters (for example, laser power; Supplementary Fig. 13) were also investigated. With increasing excitation wavelengths, the fluorescence intensity ratio between the two samples is reversed, resulting in different observed colours. Because our highly flexible nanoFlash synthesis enables multiple tunable parameters (Supplementary Fig. 14 and Supplementary Table 3), a library with thousands of 1 mm^2^ nanofilms, exhibiting fluorescent colours from violet-blue to red (Supplementary Figs. 15 and 16, and Fig. 3a, analysed in RGB channels), was established. To quantitatively reveal the connection between their performance and synthesis conditions, we introduced machine learning and the SHAP (SHapley Additive exPlanations) descriptor to the library (Supplementary Fig. 17). Seven different models (for example, random forest regression (RF) and extreme gradient boosting (XGB)) were trained, and three common property criteria (for example, coefficient of determination) were determined to validate the predictions. XGB outperformed the other six models and generated 176,640 predicted datasets to computationally extend the library with the highest reliability (Supplementary Figs. 18–20). This huge database offers great potential for optimization of SSF carbon dots to fulfil the needs in different fields, such as bioimaging, security, photocatalysis, sensors and optoelectronic devices (Supplementary Table 4).

Furthermore, the SHAP summary plot (Fig. 3b) gives details on how the fluorescence output depends on different parameters. Based on the ranking of SHAP values, the donor absorber was recognized as the most important feature for the fluorescence intensity in all three channels (Supplementary Figs. 21–23). In addition, the effect of different additives on the fluorescence intensity (Fig. 3b and Supplementary Figs. 24 and 25) is also clearly listed. For example, the addition of polyvinyl alcohol (PVA) gave enhanced red fluorescence, whereas the addition of polyethylene glycol (PEG) was detrimental. Another important part of the information in the library is that nanofilms with similar fluorescent colours have distinct micropatterns (for example, particle-like droplets, stripe-like droplets, continuous films with random defects; Fig. 3c). The shape and density of these microstructures directly influence the security level and encoding capacity of the anti-counterfeiting patterns, offering more possibilities for practical applications. The height maps and profiles (Supplementary Fig. 26) of the nanofilms show that stripe-like droplets have a higher thickness of 100–150 nm than particle-like droplets (30–80 nm) or continuous films (80–95 nm). The equivalent radius distribution of the extracted particle-like droplets follows a random Gaussian distribution with a Kolmogorov–Smirnov *P*-value of 0.007 (Fig. 3d and Supplementary Fig. 27a). Droplet orientations were detected and characterized by the autocorrelation function (Fig. 3e and Supplementary Fig. 27b). The calculated texture-aspect ratio of stripe-like droplets is 0.566, which is much higher than the reference value of 0.3 for significant preferential directions (ISO 25178), suggesting the randomness of droplet angular orientation. We can therefore conclude that these microstructures consist of one or more randomly distributed features involving the position, size and orientation of the microdroplets, which enables them to serve as PUF patterns.

## Description and authentication of PUF patterns

**Fig. 4:**
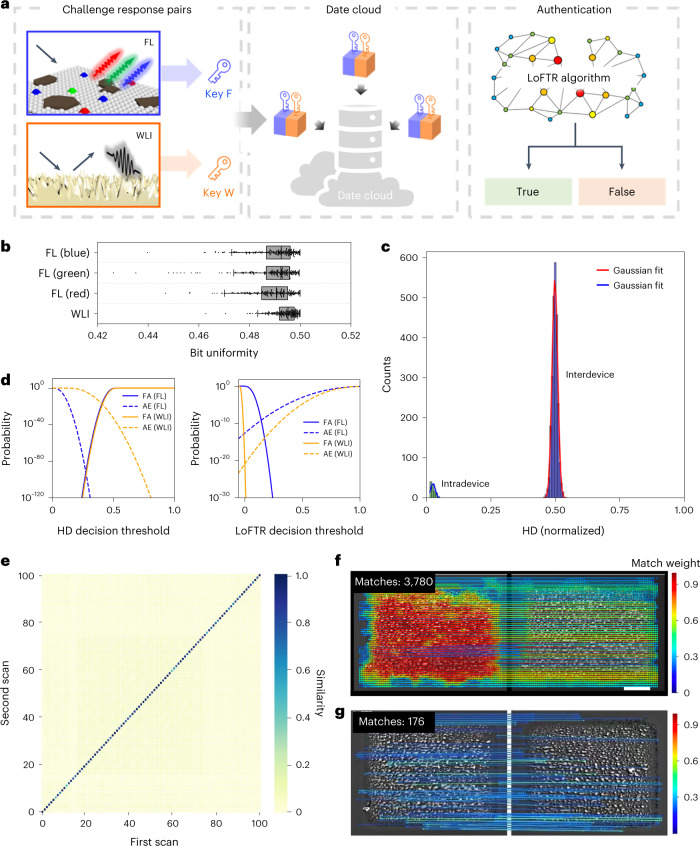
Description and authentication of PUF patterns. **a**, Workflow for PUF key generation, database initialization and authentication. **b**, Bit uniformity calculated from 100 different PUF patterns with two independent readouts (*n* = 200; centre line, median; box limits, upper and lower quartiles; whiskers, outermost data point that falls within 1.5× interquartile range; points, outliers). **c**, Device uniqueness of the PUF patterns was characterized by interdevice HD for the blue channel. The readout reproducibility (that is, the bit error rate) of the PUF patterns was characterized by the intradevice HD, where each PUF pattern was scanned twice. **d**, Cumulative distribution functions, showing the probabilities of false authentication (FA) and authentication error (AE) as a function of decision threshold for HD- and LoFTR-based authentication. **e**, Heat map of WLI similarity values (match ratio, referenced to maximum measured match number: 4,256) obtained from 100 unique PUF patterns, created using the same nanoFlash transfer parameters. **f**,**g**, Feature matching by the LoFTR algorithm for the same (**f**) and two different (**g**) PUF patterns from two independent scans. Scale bar, 200 µm. Source data

To evaluate the properties of the PUF patterns, we have performed statistical analyses on both FL and WLI scanning of the nanofilms (Fig. 4a). These two measurements do not require long readout times (Table 1) or expensive equipment. We produced 100 individual PUF patterns (each 950 × 950 µm^2^, 49 or 105 mW power, 100 or 150 mm s^−1^ scanning speed). Every sample was scanned twice with repositioning of the sample in between. The obtained data was transformed into binary signals for the Hamming distance (HD) calculations. The average bit uniformity of both FL and WLI readouts is close to the ideal value of 0.5, with a narrow standard deviation (Fig. 4b, Supplementary Fig. 28 and Table 1), confirming the high randomness. Device uniqueness and reliability are quantified by the inter- and intradevice HD^26,42^. For FL scanning, histograms of the normalized interdevice HDs centre around 0.5 (Fig. 4c), with high reliability values above 0.95 (Table 1). Similar to other height-detection measurements, each WLI readout requires an individual threshold, which makes picture processing more difficult. Thus, WLI scanning showed a reliability slightly below 0.9, which is still acceptable. The PUF patterns generated by the all-in-one nanoFlash method have high uniqueness and offer a robust response to repeated challenges.

**Table 1:** PUF parameters for fluorescence and topography characterization

Method		Scan speed	Resolution	Bit uniformity	Uniqueness	Reliability	Theoretical key space
Fluorescence	Blue		*x*,*y*: ∼2 µm	0.490 ± 0.009	0.498 ± 0.010	0.971 ± 0.012	
	Green	∼s	*z*: 16 bit intensity (AFU)	0.489 ± 0.012	0.498 ± 0.010	0.966 ± 0.014	2^105,625^ = ∼10^31,796^
	Red			0.489 ± 0.009	0.498 ± 0.009	0.970 ± 0.010	
Topography		∼s	*x*,*y*: ∼2 µm	0.494 ± 0.005	0.497 ± 0.013	0.899 ± 0.027	2^105,625^ = ∼10^31,796^
			*z*: ∼1 nm				
Integrated		∼s	*x*,*y*: ∼2 µm	0.492 ± 0.018	0.498 ± 0.021	0.934 ± 0.034	2^211,250^ = ∼10^63,593^
			*z*: 16 bit per 1 nm				

Calculated values are the mean ± s.d., integrated values are the mean with error propagation.

During the above picture processing, the edges (150 µm) of the patterns were cut off, resulting in 325 × 325 pixels for each PUF pattern. Because we have two independent readout methods (FL and WLI), the theoretical encoding capacity could reach about 10^63,593^ (Table 1), satisfying the typical criterion for a strong PUF device^43^. From the authentication results, we derived the false authentication and authentication error functions (Fig. 4d). At decision thresholds of 0.27 and 0.41 for FL and WLI, respectively, the probability of HD-based false authentication provides an estimate for the probability of cloning of a pattern of below 10^−93^ and 10^−18^. Next, the open-source algorithm LoFTR^44^ was adopted for the authentication process. We synthesized 100 individual PUF patterns with the same parameters (49 mW power, 100 mm s^−1^ scanning speed). All samples were scanned twice with FL or WLI. The LoFTR compared every combination of two pictures and recorded the number of matches for similarity analysis and correlation calculations (Fig. 4e–g and Supplementary Fig. 29). A clear separation of high intracorrelation (WLI, 87.0%; FL, 88.5%) from lower intercorrelation (WLI, 3.8%; FL, 0.3%) verified that each PUF pattern exhibits a distinct microstructure (Supplementary Table 5). At decision thresholds of 0.15 and 0.01 (Fig. 4d), the LoFTR-based probability of cloning is estimated to be below 10^−9^ and 10^−20^ for FL and WLI, respectively. In addition, the readout from different resolutions (1, 5 and 10 µm per pixel) and scanners (Molecular Devices Genepix 4000B, Innopsys Innoscan 1100AL) was also analysed (Supplementary Fig. 30). A sufficiently large gap between inter- and intracorrelation was always observed, indicating high reliability for practical authentication. An exception is the dramatically dropped similarity between the pictures obtained from 1 µm per pixel and other resolutions (5 or 10 µm per pixel). This can be solved by registering multiple resolutions in the data cloud, to avoid false-negative results.

## Combining PUF microstructures with macroscopic patterns

**Fig. 5:**
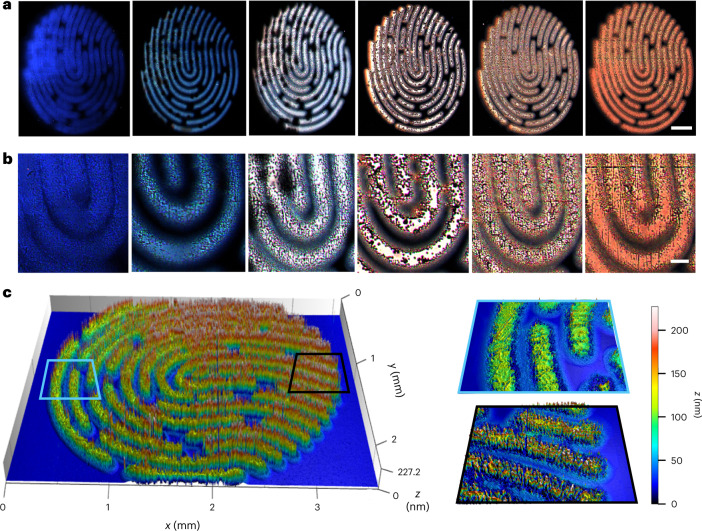
Artificial fingerprints with micromorphology and nanothickness. **a**, Fingerprint patterns with different solid-state fluorescence colours were generated with parameters from the extended library. Scale bar, 500 µm. Detailed synthesis parameters can be found in Supplementary Table 6. **b**, Magnification showing the micromorphology of the patterns. Scale bar, 100 µm. **c**, 3D height map of the fingerprint pattern shown in the rightmost panel of **a**. Original fingerprint image, credit: iStock.com/330_librarians.

Combining the nanoFlash process with a defined macroscopic pattern can add extra encryption for anti-counterfeiting labels, especially when the macropattern has been designed to selectively show or hide specific information under different readout methods^18,45^. We planted PUF structures in an artificial fingerprint pattern (Fig. 5a) to visualize the independent microstructures in the fluorescence (Fig. 5b) and topography (Fig. 5c) channels. The synthesis parameters for the fluorescent fingerprint patterns were derived from the library (Supplementary Table 6) and high colour reproducibility was observed. The height maps of the fingerprints could serve as an additional PUF feature to the fluorescence microstructures, making the patterns immune to attacks such as nanomoulding. Another scanning after 2 months revealed that the nanothickness maps remained unchanged, suggesting high stability (Supplementary Figs. 31 and 32). Restricting the nanoFlash process to a defined macropattern does not change the micro-/nanoscopic nature and general properties of PUF structures^46^. However, it could cause a potential bias during authentication because the defined macropattern is much more easily recognized by algorithms in comparison to the microstructures. We analysed the inter- and intracorrelation of different fingerprint patterns, which were scanned directly or after 2 months (Supplementary Fig. 33). Although the intracorrelation is sometimes lower than those from the simple square PUF patterns (Fig. 4e), the distinct difference between the intra- and intercorrelation makes it sufficient for a reliable authentication process.

## Conclusions

The nanoFlash synthesis method we have described is an all-in-one strategy in that it generates SSF CD films in situ and simultaneously prints unclonable nano-/microstructures. During conventional solvent-free synthesis in an oven, large carbon flakes, formed by overcarbonization, cause fluorescence quenching. Our approach avoids this problem by precisely controlling the heating process in milliseconds. The generated CDs are dispersed in the non-reacted monosaccharide precursor, which greatly reduces Förster resonance energy transfer. Therefore, the CD nanofilms show quenching-resistant properties. We generated a nanofilm library (1,920 experimental datasets) with tunable SSF from violet-blue to red and different microstructures. The distinct randomness of the microstructures allows the nanofilms to be used as PUF patterns. Near-ideal bit uniformity, high uniqueness and excellent reliability were observed, suggesting these films have excellent PUF performance. Furthermore, the combination of independent fluorescence and topography readout greatly improves the security level of the PUF patterns, making them immune to attacks by nanomoulding and nanolithography. Through the open-source algorithm LoFTR, a significant gap between the intra- and intercorrelation promises precise authentication regardless of the readout methods (FL or WLI) that end-users choose. In conclusion, we also note that our technique could be useful for future syntheses of colour conversion layers, quenching-resistant (organic) light-emitting diodes or sensitive bio-/chemosensors.

## Methods

### Solvent-free synthesis process

#### Film preparation

Typically, 1 g d-(+)-glucosamine hydrochloride (99%, Sigma) was dissolved in 3 ml H_2_O. A clean glass slide was covered by a scotch tape (3M company) frame with a defined 1.5 cm × 1.5 cm area (Supplementary Fig. 1). We deposited 300 µl of the solution on the surface of the glass and dried it in an oven at 50 °C for around 30 min. Afterwards, the tape was removed and the film was placed in an air oven for thermal annealing.

#### Characterization

The annealed films were removed from the surface and dissolved with 2 ml H_2_O in Eppendorf tubes. After centrifuging at 9,391*g* (10,000 r.p.m.) for 10 min, the supernatant was filtered through a 0.45 µm hydrophilic filter (CHROMAFIL Xtra, 13 mm). The obtained solution was used for the following characterizations. Ultraviolet–visible spectra were recorded with a UV-1900 spectrometer (Shimadzu). Fluorescence spectra of CDs were measured using a microplate reader (SpectraMax M5, Molecular Devices). Mass spectra were recorded using an HPLC-System Series 1100 coupled with ESI-single quadrupole from Agilent. NMR spectra were obtained on an AscendTM 400 spectrometer (400 MHz, Bruker) at 298 K, and are reported in ppm relative to the residual solvent peaks. For ^1^H and ^13^C spectra, the films were directly dissolved in 600 µl D_2_O (Sigma) before centrifugation and filtration. The TEM study was performed using a double Cs corrected JEOL JEM-ARM200F (scanning) transmission electron microscope operated at 80 kV and equipped with a cold-field emission gun. Annular dark-field scanning transmission electron microscopy images were collected at a probe convergence semi-angle of 25 mrad. Atomic force microscopy measurements were recorded with a JPK Bruker NanoWizard4 instrument in a.c. (tapping) mode.

#### Colour coordinate calculation

The colour coordinates of fluorescence were presented in CIE 1931 colour space according to the standard of the International Commission on Illumination. In a typical test, the emission intensities (Em) of material were collected every 5 nm in the wavelength range between (excitation wavelength + 20) nm and 700 nm to avoid the influence of the excitation resource. Multiple emission spectra were obtained under different excitation wavelengths in the range between 300 and 600 nm (20 nm steps), finally producing a 2D fluorescence spectrum for the studied material. Before calculation, all Em were normalized by the highest Em in the 2D fluorescence spectrum and only major emission spectra were taken into consideration (normalized Em_max_ > 0.5).

#### Temperature diffusion simulation

The temperature diffusion during the laser irradiation process was simulated by ANSYS with a steady-state thermal analysis system. The geometry model was constructed using 3dsMax as a file in SAT format. Standard engineering data were used, including the thermal conductivity of industrial glass, haematite and glucose. The model was automatically meshed by the MultiZone method with no suppression. The initial temperature of the irradiated laser spot was set to 25 °C (environment temperature), the final point was set to 1,000 °C. Thermal convection of all faces of the model were taken into consideration. The simulation lasted for 1 s.

### nanoFlash approach

#### Preparation of donor slides

##### Absorber layer

The haematite films were generated based on our previous work^47^. Briefly, two solutions need to be prepared and mixed: 125 mg of PVA (average *M*_r_ ≈ 9,000–10,000, Sigma) and 125 mg of Fe(NO_3_)_3_·9H_2_O (98%, Acros) in 250 µl of double-distilled H_2_O; 250 mg of PEG (average *M*_r_ ≈ 20,000, Sigma) and 250 mg of Fe(NO_3_)_3_·9H_2_O in 250 µl of methanol. Then, the solution was spin-coated onto a clean glass slide at 70 r.p.s. and the slide was annealed in an air oven at 500 °C for 3 h. After cooling down, the final haematite layer was obtained. For the CuO absorber layer, we dissolved 0.175 g Cu(NO_3_)_2_·*x*H_2_O (99%, Acros) and 0.175 g PVA (average *M*_r_ ≈ 9,000–10,000, Sigma) in 0.5 ml of H_2_O and spin-coated the solution onto a glass slide at 70 r.p.s. Then, the slide was annealed at 500 °C for 3 h in an air oven. After cooling down, the final CuO layer was obtained as a black film.

##### Material layer

First, 25 mg (or 50, 100, 150 mg, depending on the specific recipes) of d-(+)-glucose (Merck) was dissolved in 500 µl of H_2_O. We spin-coated the solution onto the absorber layer at 70 r.p.s. to obtain a homogeneous material layer. By replacing d-glucose with d-(+)-glucosamine hydrochloride (99%, Sigma), d-galactose (Carbosynth) or *N*-acetylglucosamine (99%, Sigma), material layers with other precursors were obtained.

#### Printing process

After cleaning the back side of the donor slide, we placed it on top of a clean glass which served as the acceptor slide. During the printing process, we use a 200 mW TOPTICA iBeam smart 488-S laser (488 nm, TOPTICA Photonics), which is passed through a 1:10 beam expander and a Racoon 11 laser scan head (ARGES) equipped with an f-Theta lens. The generated patterns were generally characterized by fluorescent images with a high-resolution fluorescence scanner (Innopsys, Innoscan 1100AL) at a pixel resolution of 5 µm and a scanning speed of 25 lines per second. The red channel was excited at 635 nm with an emission filter of 680/42; the green channel was excited at 532 nm with an emission filter of 605/15; the blue channel was excited at 488 nm with an emission filter of 520/5. Detection was performed with a gain factor of 5 and low laser power. The thickness information was gained by vertical scanning interferometry with a smartWLI compact (Gesellschaft für Bild- und Signalverarbeitung). For morphology analysis, scanning electron microscopy was carried out using a Zeiss LEO 1550 microscope equipped with a field emission gun and with an Oxford Instruments X-MAX SDD X-ray energy-dispersive detector (detection area, 80 mm^2^); images were recorded at 3 kV.

#### Library preparation

The precursor type and concentration, absorber material and thickness, and additives are the factors that could be tuned during the preparation of donor slides. Different laser foci were achieved by adjusting the distance of the sample stage from the scan head. In the focus plane, the laser spot was measured with a 1/*e*^2^ diameter of 18 µm as reported previously^48^. Other planes, which result in larger laser spots, are defined as the low focus area. The scanning speed, laser power and printing mode (optimized line scanning versus bitmap scanning) are controlled by the laser scanning system.

### Machine learning

#### Extraction of the dataset

The fluorescent films generated in the library were used to obtain the dataset. The fluorescence intensity from three channels was read from CSV files exported by the high-resolution scanner and the mean value of the intensity was recorded in the dataset. Data cleaning was conducted to fix or remove incorrect, duplicate or incomplete data. After obtaining a dataset suitable for training, the dataset was then shuffled and divided into the training set and test set at a ratio of 4:1. We used one-shot encoding for categoric features, which expanded the value of discrete features to Euclidean space and solved the problem that the model could not directly deal with categoric features.

#### Machine learning models

We introduced different regression models to predict the fluorescence intensity. Scikit-Learn was used to obtain the algorithms of support vector machine regression (SVR), multilayer perceptron (MLP), *k*-nearest neighbours regression (KNN), polynomial regression (PR), decision tree regression (DT) and random forest regression (RF). The construction of XGB was performed using another independent library. Before the SVR, PR, KNN and MLP training, we normalized the features so that all of the variables are in the same range. Normalization also accelerated the gradient descent to find the optimal solution. For tree-based models (DT, RF and XGB), this step was not necessary. The repeated *k*-fold cross-validation procedure with 5 folds and 10 repeats was used to evaluate each algorithm. Importantly, all algorithms were configured with the same random seed to ensure that the same splits to the training data are performed, so each algorithm was precisely evaluated in the same way. The optimal hyperparameters for models were found by grid-search.

#### LoFTR algorithm

The LoFTR algorithm is a framework for image feature matching. Instead of performing image feature detection, description and matching one by one sequentially, it first establishes a pixel-wise dense match and refines the matches later. In contrast to traditional methods that utilize a cost volume to search corresponding matches, the framework applies self- and cross-attention layers from its Transformer model to obtain feature descriptors on both images. The global receptive field provided by Transformer enables the LoFTR algorithm to produce dense matches, even in low-texture areas, where traditional feature detectors usually struggle to produce repeatable interest points. Furthermore, the framework model is pretrained on indoor and outdoor datasets to detect the kind of image being analysed, with features such as self-attention. Hence, LoFTR outperforms other state-of-the-art methods. The LoFTR module uses self- and cross-attention layers in Transformers to transform the local features to be context- and position-dependent, which is crucial for LoFTR to obtain high-quality matches on indistinctive regions with low-texture or repetitive patterns. For image pair (*I*^A^, *I*^B^), their similarity is defined by the number of matched features:

$${\mathrm{similarity}}({I}^{{\mathrm{A}}},{I}^{{\mathrm{B}}})=\frac{{N}_{{\mathrm{matches}}}({I}^{{\mathrm{A}}},{I}^{{\mathrm{B}}})}{{N}_{{\mathrm{features}}}},$$

where *N*_matches_(*I*^A^, *I*^B^) is the number of matched features and *N*_features_ is the number of extracted features. The LoFTR algorithm extracts 4,800 features in total; however, for the border area, a mask has been applied for practical analysis. Therefore, the maximum number of possible extracted features is 4,256. The resulting colourmap shows the matching probability *P*_c_. In the case of dual-softmax, it is obtained by

$${P}_{{\mathrm{c}}}\left(i,j\right)={{\mathrm{softmax}}(S\left(i,\cdot \right))}_{j}\cdot {{\mathrm{softmax}}(S\left(\cdot ,j\right))}_{i}.$$

The score matrix *S* between the transformed features is calculated by

$$S(i,j)=\frac{1}{\tau }\cdot \left\langle {\widetilde{F}}_{{\mathrm{tr}}}^{{\mathrm{A}}}(i){\widetilde{F}}_{{\mathrm{tr}}}^{{\mathrm{B}}}(\,j)\right\rangle ,$$

where ${\widetilde{F}}_{\mathrm{tr}}^{\mathrm{A}}$ and ${\widetilde{F}}_{\mathrm{tr}}^{\mathrm{B}}$ are the added features that enter the LoFTR module for processing.

### Characterization of PUF properties

The texture-aspect ratio was calculated based on the following equation:

$$\begin{array}{l}{\rm{texture}}\mbox{-}{\rm{aspect}}\,{\rm{ratio}}=\frac{\mathop{{\rm{min}}}\limits_{{tx},{ty}\in R}\sqrt{{{tx}}^{2}+{{ty}}^{2}}}{\mathop{{\rm{max}}}\limits_{{tx},{ty}\in R}\sqrt{{{tx}}^{2}+{{ty}}^{2}}}\\{\mathrm{where}}\,R=\{\left({tx},{ty}\right):{\mathrm{ACF}}\left({tx},{ty}\right)\le s\}\end{array}$$

where the texture-aspect ratio is the ratio of the minimum to maximum horizontal distance of the central lobe (generated by thresholding the central normalized autocorrelation peak) of the autocorrelation function ACF(*tx*,*ty*). The minimum horizontal distance is the fastest decay to a specified value *s* (standard default setting *s* = 0.2) and the maximum horizontal distance is the slowest decay to *s*.

To evaluate the properties of the PUF patterns, the data were transformed into binary signals by setting the median value as a predetermined threshold for the following analysis. The bit uniformity estimates the distribution of logic-0 and logic-1 in PUF pattern responses. It can be calculated using the following equation:

$${\mathrm{bit}}\,{\mathrm{uniformity}}=\,\frac{1}{k}\,\mathop{\sum }\limits_{i=1}^{k}{R}_{i}$$

where *R*_*i*_ is the *i*th binary bit of the PUF pattern and *k* is the total number of PUF patterns.

The uniqueness between any two PUF patterns can be defined as:

$${\mathrm{uniqueness}}=\,\frac{2}{k(k-1)}\mathop{\sum }\limits_{i=1}^{k-1}\mathop{\sum }\limits_{j=i+1}^{k}\frac{\mathrm{HD}\big({R}_{i}\left(n\right),\,{R}_{j}\left(n\right)\big)}{n}$$

where *R*_*i*_(*n*) and *R*_*j*_(*n*) are the *n*-bit responses of the *i*th and *j*th PUF patterns, respectively, and *k* is the total number of PUF patterns.

All PUF patterns were scanned twice and the reliability was evaluated using the following equation:

$${\mathrm{reliability}}=1-\frac{1}{k}\mathop{\sum }\limits_{i=1}^{k}\frac{1}{T}\mathop{\sum }\limits_{l=0}^{T}\frac{\mathrm{HD}\big({R}_{i}^{0}\left(n\right),{R}_{i}^{l}\left(n\right)\big)\,}{n}$$

where ${R}_{i}^{l}\left(n\right)$ is the *n*-bit response from the *i*th PUF at the *l*th trial, *T* is the number of trials and *k* is the total number of PUF patterns.

### Time-resolved photoluminescence spectroscopy and fluorescence anisotropy analysis

Time-resolved photoluminescence spectroscopy showed the lifetime of the excited state for our typically generated materials (dissolved nanoFlash film) to be 8.7 × 10^−8^ s at 420 nm emission under 365 nm excitation. Next, fluorescence anisotropy measurements were performed to visualize the Brownian motion and estimate the size of the generated fluorescent materials^49,50^. Based on the transformed Perrin equation, the rotational correlation time (*Φ*_r_) can be calculated:

$$\frac{{r}_{0}}{r}=1+\frac{\tau }{{\varPhi }_{\mathrm{r}}},$$

where *r* is the observed anisotropy, *r*_0_ is the intrinsic anisotropy of the molecule and *τ* is the fluorescence lifetime. According to the parallel fluorescence intensity (*I*_||_ = 2,043) and perpendicular fluorescence intensity (*I*_⊥_ = 1527), *r* was calculated to be 0.1, while *r*_0_ had a maximum value of 0.4 with parallel excitation and emission dipoles, with *Φ*_r_ = 2.9 × 10^−8^ s. This can be inserted into the Debye–Einstein–Stokes equation:

$${\varPhi }_{\mathrm{r}}=\frac{\mu V}{{K}_{\mathrm{B}}T},$$

where *μ* and *V* are the viscosity and volume of the tested sample, respectively, *K*_B_ is the Boltzmann constant and *T* is the temperature for the test in Kelvin. Therefore, the volume of the generated materials was calculated to be 1.2 × 10^−25^ m^3^. By simplifying the particle to a standard sphere, the generated materials had an estimated average diameter of about 3.1 nm.

## Online content

Any methods, additional references, Nature Portfolio reporting summaries, source data, extended data, supplementary information, acknowledgements, peer review information; details of author contributions and competing interests; and statements of data and code availability are available at 10.1038/s41565-023-01405-3.

## Supplementary information

Supplementary Information Supplementary Figs. 1–33, Supplementary Tables 1–6 and description of Supplementary Movie 1

Supplementary Code Comparing different machine learning models for fluorescence colour prediction.

Supplementary Video 1 Example transfer of a pattern.

## Data Availability

All data are available in the manuscript or the supplementary materials. Source data are provided with this paper.

## Source data

Source Data Fig. 2 Contains: Figure 2bcd source data.xlsx. Figure 2e_blue source data.png. Figure 2e_green source data.png. Figure 2e_red source data.png. (b, c, d) Original data for XPS plots, (e) three fluorescence channel images for correlation analysis.
Source Data Fig. 3 (b) Original median fluorescence data acquired from library in respect to the synthesis parameters, (d, e) source data acquired from analysis/measurement Mountains Map Software.
Source Data Fig. 4 (b) Original HD data, (c) inter- and intradevice HD, (e) similarity map from LoFTR on WLI measurement data.

## Code availability

The software code is part of the Supplementary Information.
