# Supplementary material for: An all-in-one nanoprinting approach for the synthesis of a nanofilm library for unclonable anti-counterfeiting applications
Source: Nat Nanotechnol. 2023 Jun 5;18(9):1027–35. doi: 10.1038/s41565-023-01405-3 (PMC10501905; doi:10.1038/s41565-023-01405-3)
Supplement: Supplementary file 1 — Supplementary Figs. 1–33, Supplementary Tables 1–6 and description of Supplementary Movie 1 [file 41565_2023_1405_MOESM1_ESM.pdf]

nature nanotechnology

Article https://doi.org/10.1038/s41565-023-01405-3

# An all-in-one nanoprinting approach for the synthesis of a nanofilm library for unclonable anti-counterfeiting applications

In the format provided by the authors and unedited

## Table of contents

**Supplementary Figure 1.** Film preparation for solvent-free synthesis process and UV-vis absorption spectra.

**Supplementary Figure 2.** 2D fluorescence spectra and calculated color coordinates of oven annealed films.

**Supplementary Figure 3.** $^1$H NMR spectra of fluorescent carbon dots obtained by solvent-free synthesis.

**Supplementary Figure 4.** Mass spectrometry of the films annealed at 140 °C for 1 h.

**Supplementary Figure 5.** The laser setup for the nanoprinting-assisted flash synthesis (nanoFlash) approach.

**Supplementary Figure 6.** Scanning electron microscopy imaging and fluorescence scanning of films.

**Supplementary Figure 7.** Polymer coating for water resistance.

**Supplementary Figure 8.** High-resolution XPS binding energy spectrum of the precursor film.

**Supplementary Figure 9.** Additional characterization of carbon dots.

**Supplementary Figure 10.** NanoFlash synthesis directly on a TEM grid.

**Supplementary Figure 11.** Investigation of temperature distribution in axial direction.

**Supplementary Figure 12.** 2d fluorescence spectra of carbon dot films (3d map).

**Supplementary Figure 13.** Fluorescence spectra of two carbon dot films under different excitation wavelengths.

**Supplementary Figure 14.** Machine learning was introduced to extend the library from ~2k (experimental) to ~176k (experimental + predicted) datasets.

**Supplementary Figure 15.** Fluorescent film library achieved by nanoFlash synthesis.

**Supplementary Figure 16.** Different laser powers and scanning speeds in each section of fluorescent film library.

**Supplementary Figure 17.** Scheme of the machine learning process.

**Supplementary Figure 18.** Determination coefficient ($R^2$) of different models.

**Supplementary Figure 19.** Root mean squared error (RMSE) of different models.

**Supplementary Figure 20.** Mean absolute error (MAE) of different models.

**Supplementary Figure 21.** SHAP feature importance for red channel.

**Supplementary Figure 22.** SHAP feature importance for green channel.

**Supplementary Figure 23.** SHAP feature for blue channel.

**Supplementary Figure 24.** SHAP summary plot in XGB model for green fluorescence.

**Supplementary Figure 25.** SHAP summary plot in XGB model for blue fluorescence.

**Supplementary Figure 26.** Height maps and profiles of PUF patterns.

**Supplementary Figure 27.** Two PUF patterns generated with different laser parameters.

**Supplementary Figure 28.** Device uniqueness of the PUF patterns.

**Supplementary Figure 29.** Heat map of FL similarity values.

**Supplementary Figure 30.** Investigation of the practical fluorescence authentication.

**Supplementary Figure 31.** 2D height map of the first 3 fingerprints in Fig. 5 directly after printing and 2 months after printing.

**Supplementary Figure 32.** 2D height map of the last 3 fingerprints in Fig. 5 directly after printing and 2 months after printing.

**Supplementary Figure 33.** Similarity analysis of topography patterns by LoFTR algorithm.

**Supplementary Movie 1.** Real-time transfer of a PUF pattern with magnification of the printed pattern.

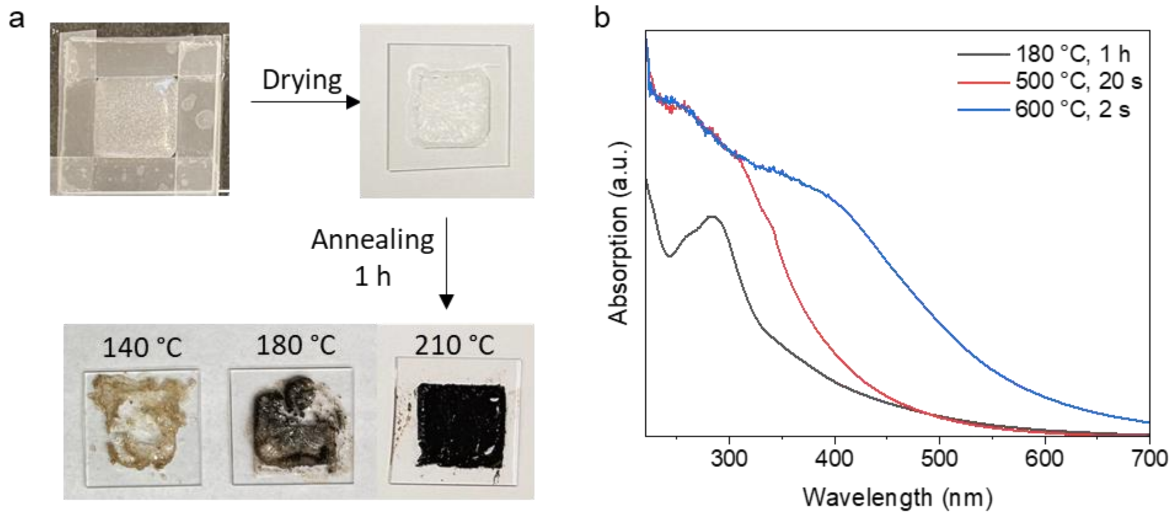


**Supplementary Figure 1.** (a) Film preparation for solvent-free synthesis process and the following annealing treatment. (b) UV-vis absorption spectra of fluorescent carbon dots obtained by solvent-free synthesis.

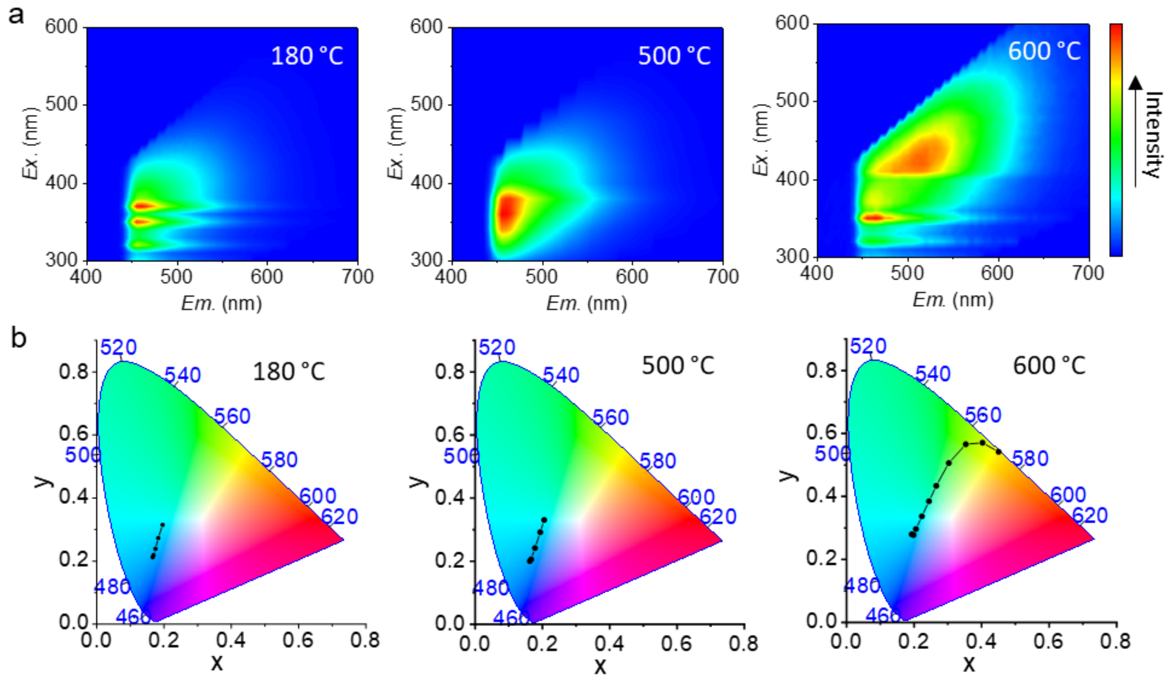


**Supplementary Figure 2.** (a) 2D fluorescence spectra and (b) calculated color coordinates (Commission internationale de l'éclairage standard) of films annealed at 180 °C for 1 h, 500 °C for 20 s, and 600 °C for 2 s.

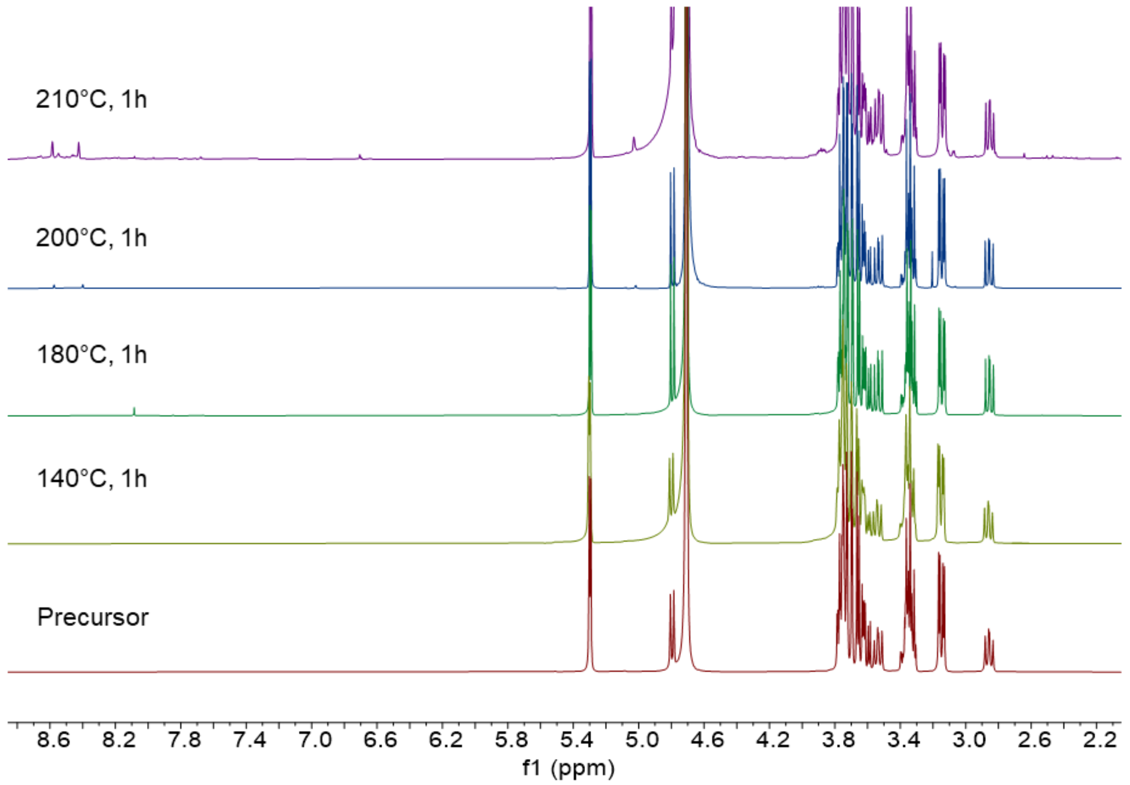


**Supplementary Figure 3. $^1H$ NMR spectra of fluorescent carbon dots obtained by solvent-free synthesis.** The precursor is D-(+)-Glucosamine hydrochloride.

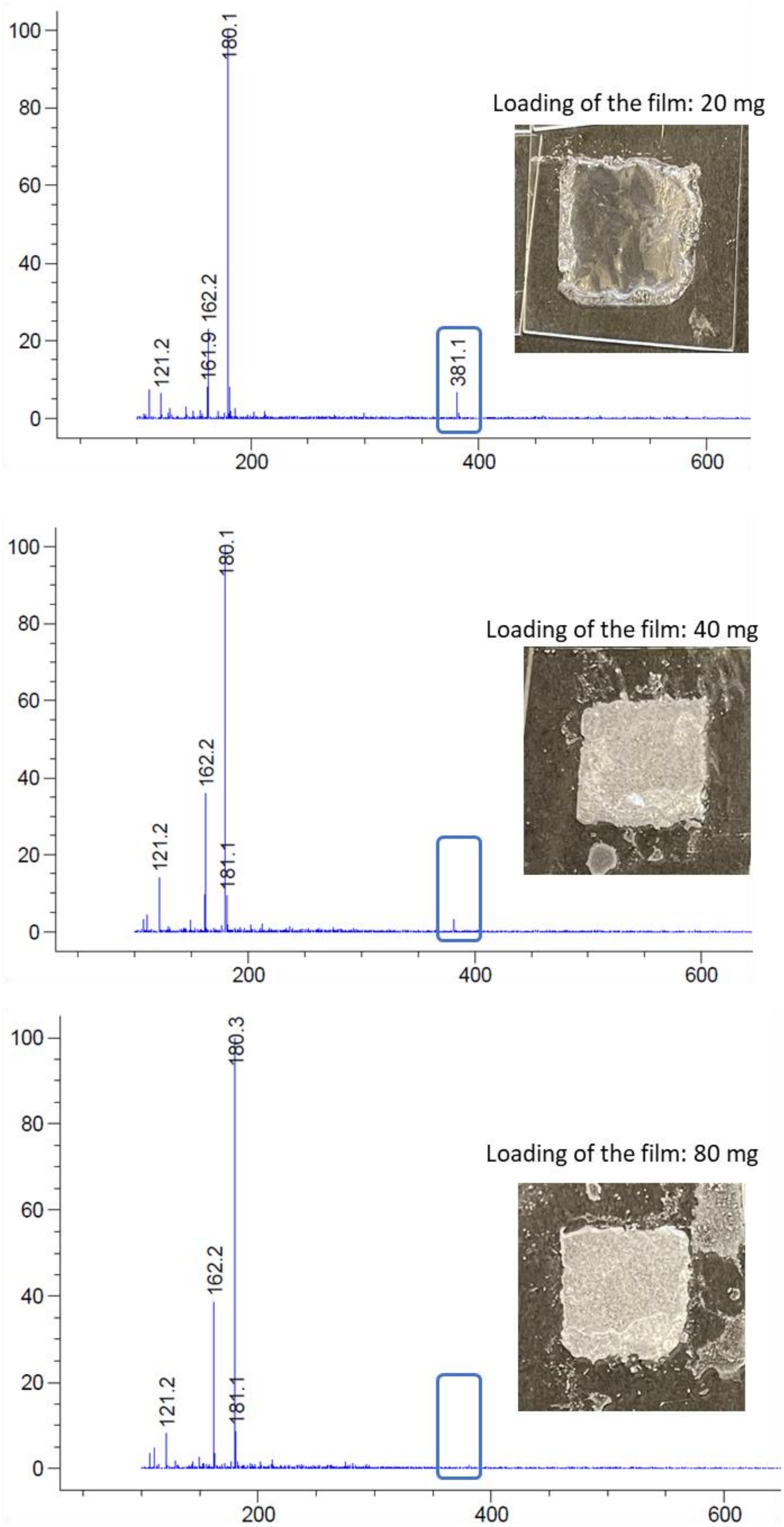


**Supplementary Figure 4. Mass spectrometry of the films annealed at 140 °C for 1 h.** All the films are the same size: 1.5 cm x 1.5 cm. Different loading was achieved by different concentrations of the precursor solution.

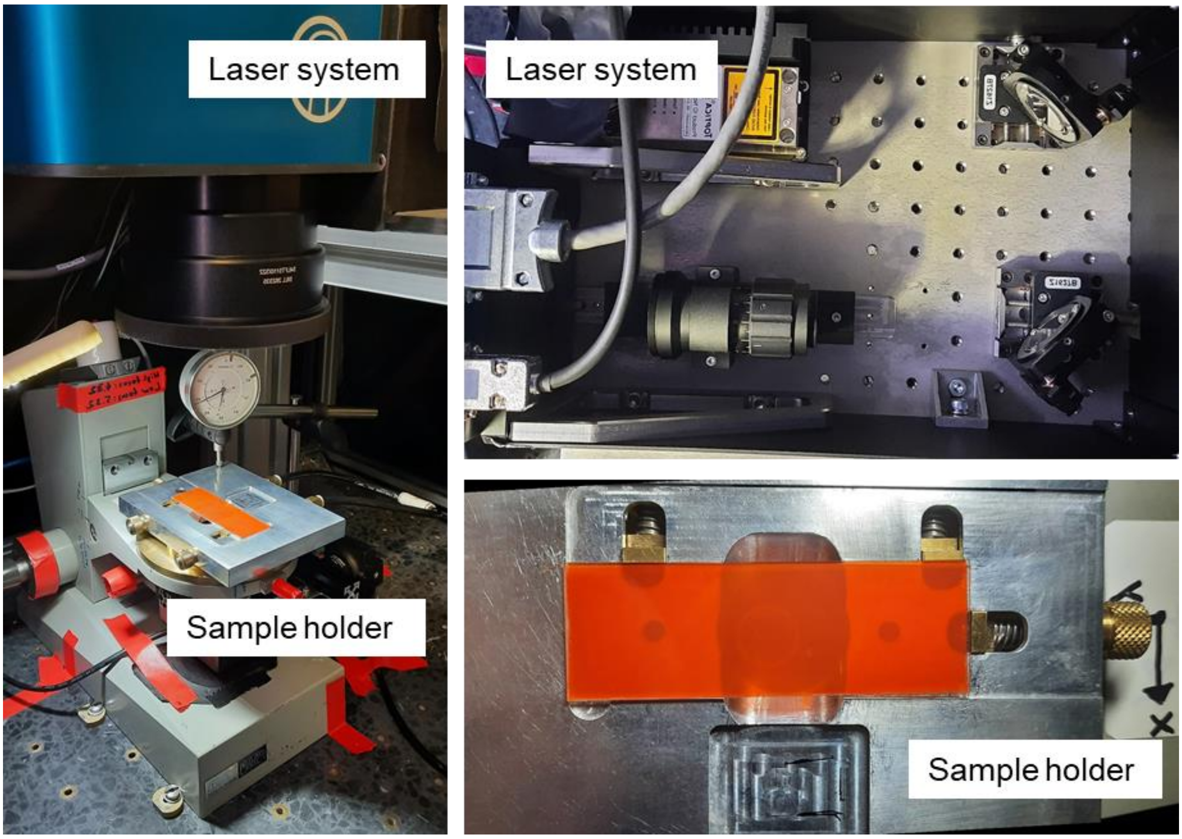


**Supplementary Figure 5. The laser setup for the nanoprinting-assisted flash synthesis (nanoFlash) approach.** The laser rapidly heats, melts, and transfers the donor material.

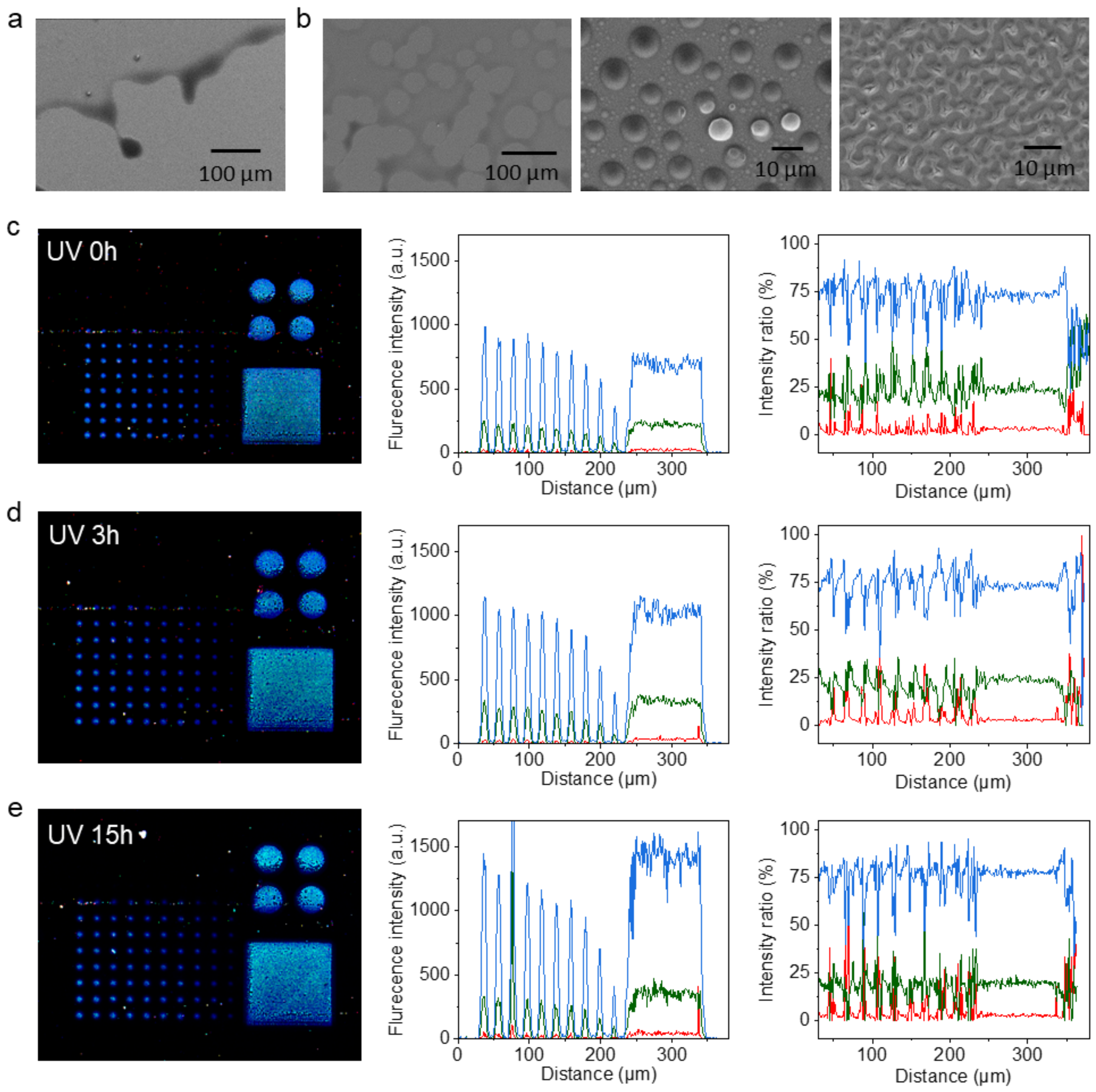


**Supplementary Figure 6.** Scanning electron microscopy imaging of (a) precursor film and (b) transferred areas. The fluorescence scanning and intensity profiles of a printed pattern (c) without UV treatment, (d) UV treatment for 3 h, (e) UV treatment for 15 h.

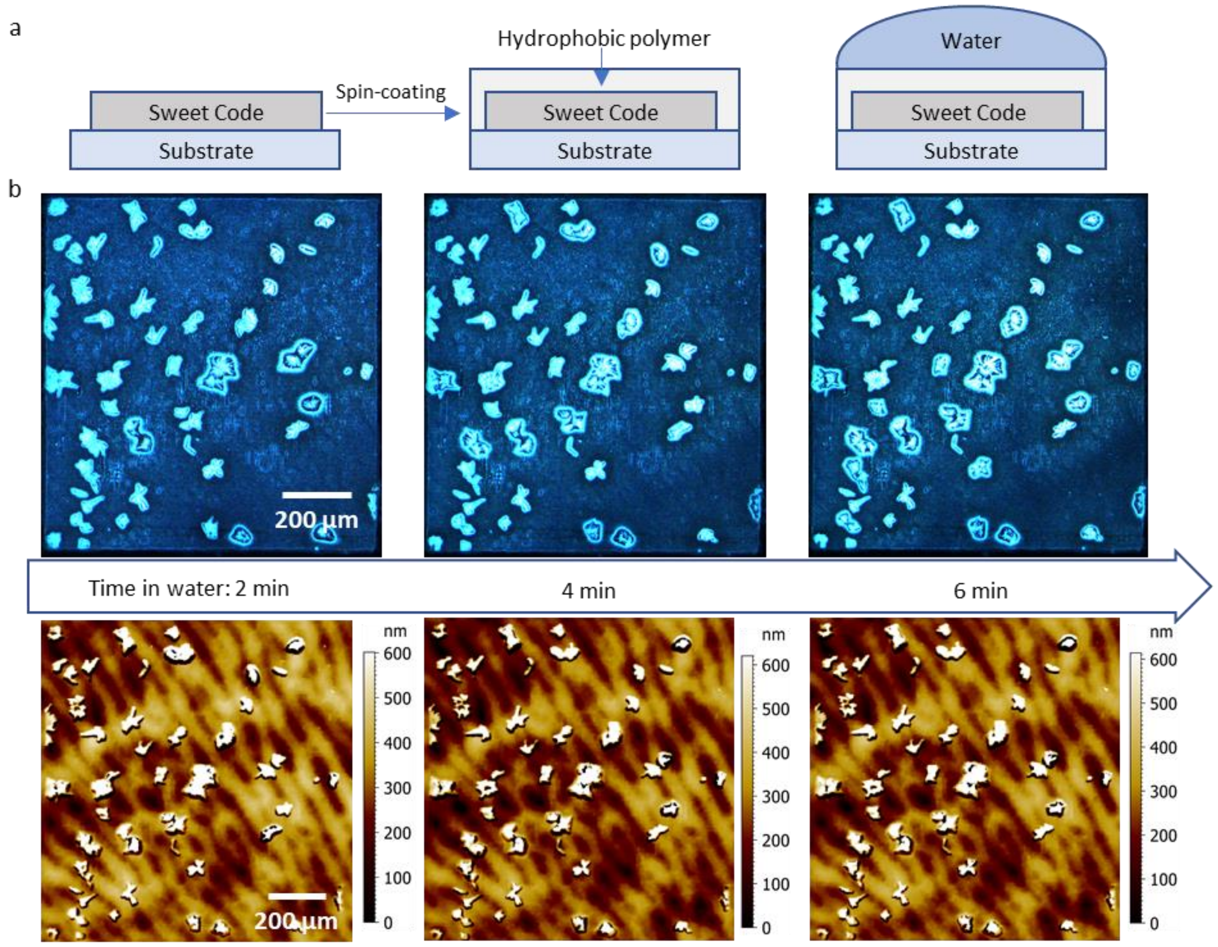


**Supplementary Figure 7. Polymer coating for water resistance.** (a) A thin hydrophobic polymer layer was introduced on the top of the Sweet Code nanopatterns by spin coating. Then, a water droplet was placed on the protected Sweet Code. (b) The pattern was scanned by fluorescence (top) and white light interferometry (bottom). The recipe of the protective polymer solution is 50 mg PMMA, 50 mg PS in 500 µl DCM, which was spin-coated at the speed of 40 rpm.

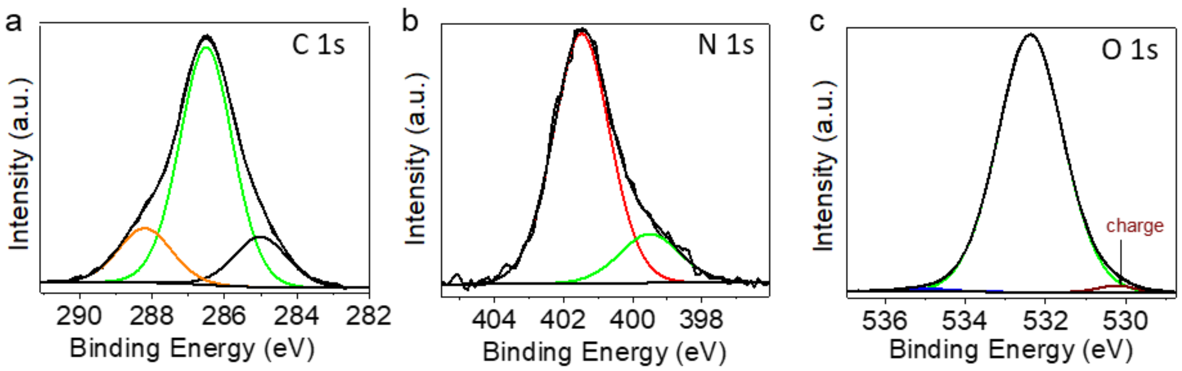


**Supplementary Figure 8. High-resolution (a) C 1s, (b) N 1s, and (c) O 1s XPS binding energy spectrum of the precursor film.** Preparation by spin-coating a pure precursor solution (glucosamine hydrochloride) on a glass substrate.

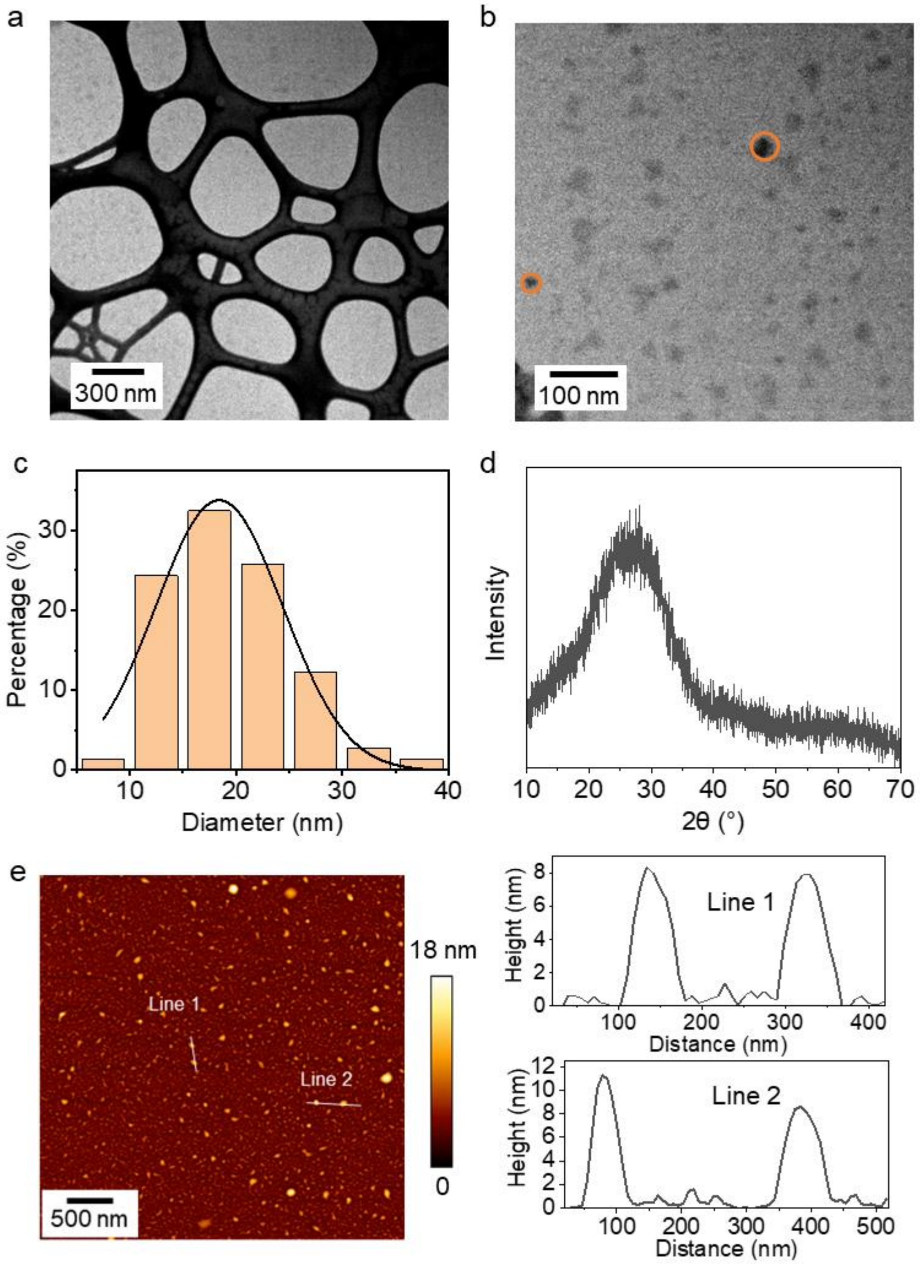


**Supplementary Figure 9. Additional characterization of carbon dots.** (a, b) Bright-field scanning transmission electron microscopy images and (c) size distribution of nanoFlash pre-synthesized material, deposited onto a TEM grid by drop casting. (d) XRD patterns of a nanoFlash generated film. (e) AFM image and the corresponding height profiles, deposited by drop casting of nanoFlash pre-synthesized material onto a silica substrate. Due to typical tip convolution effects of AFM measurements, only the height is considered as the detected particle size.

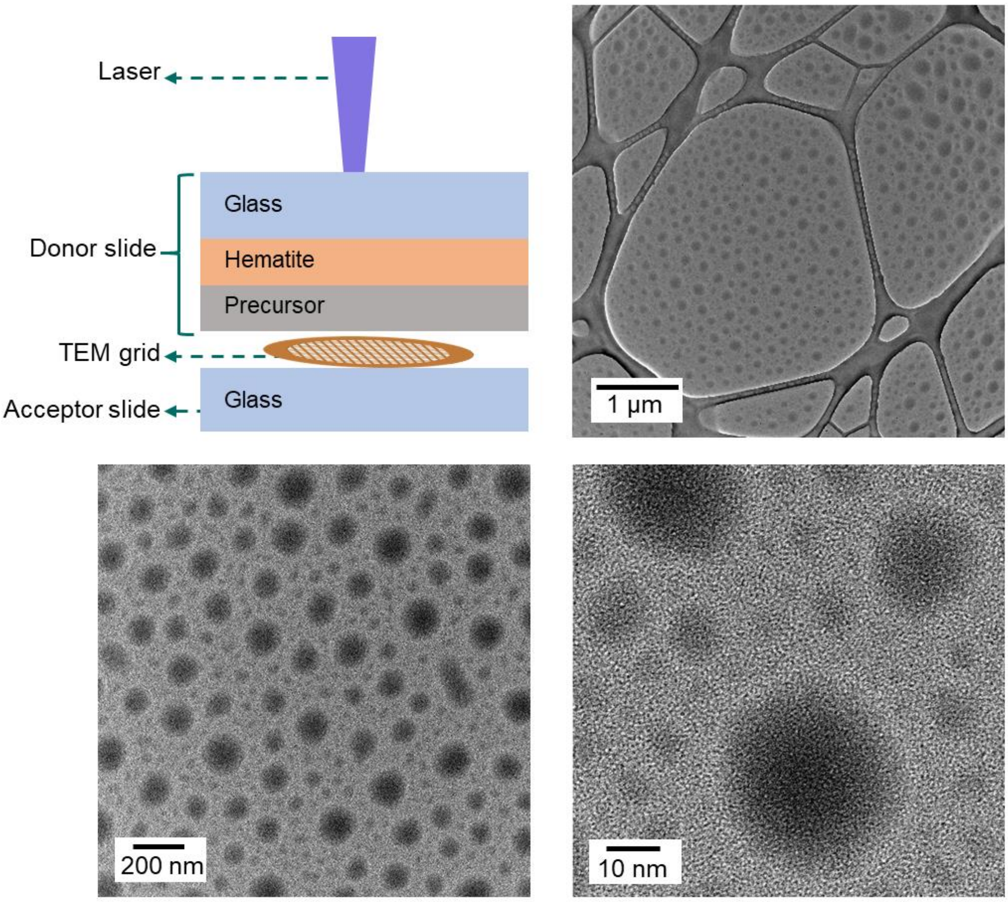


**Supplementary Figure 10.** NanoFlash synthesis directly on a TEM grid and subsequent measurement**.** No crystalline carbon species were detected.

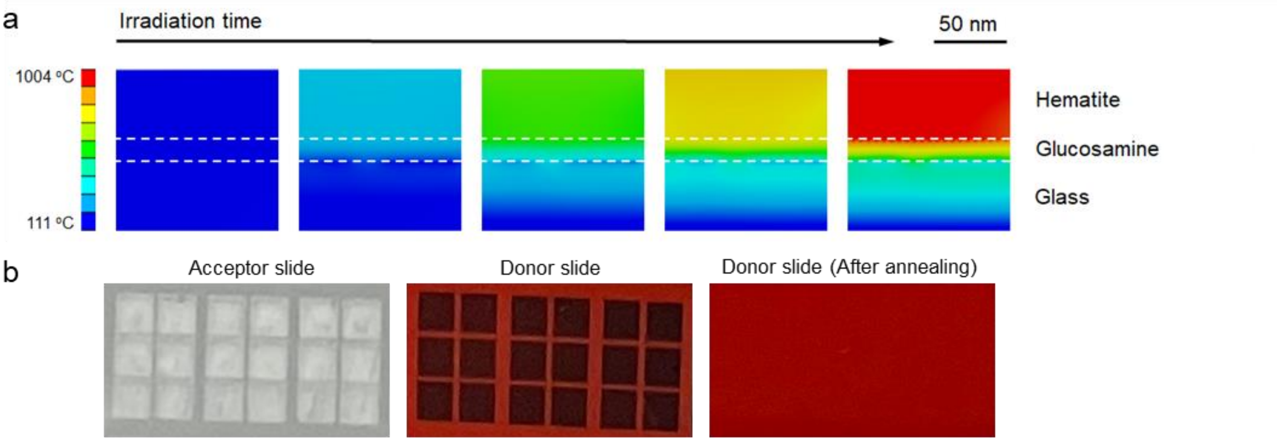


**Supplementary Figure 11. Investigation of temperature distribution in axial direction.** (a) Ideal heat transfer model (thermal diffusion), containing hematite (480 nm), glucosamine (160 nm), and glass acceptor layers. (b) Photographs of the acceptor and donor slides. The same donor slide was annealed at 500 °C for 10 min in air oven.

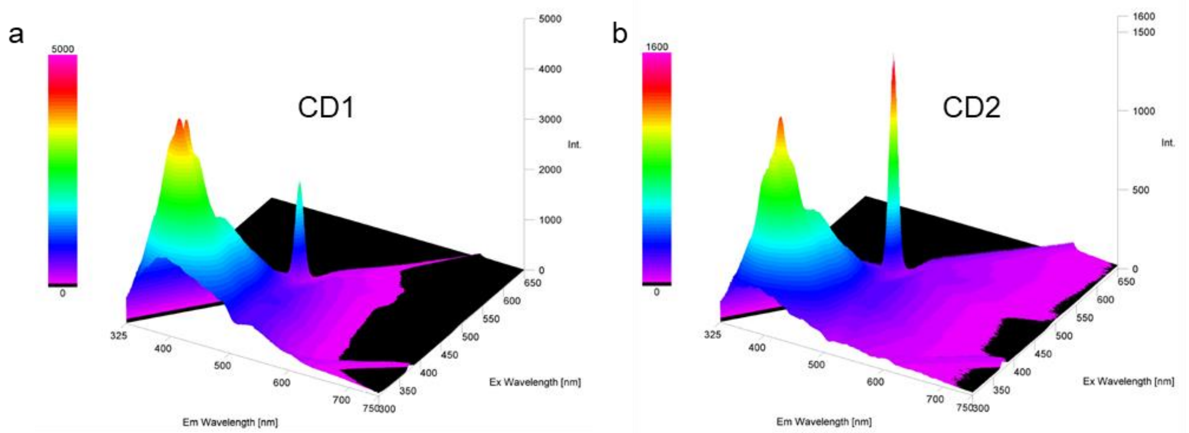


**Supplementary Figure 12. 2d fluorescence spectra of carbon dot films (3d map).** Parameters for the preparation of the CD1 film: 100 mg/ml glucosamine hydrochloride, 40% laser power, 150 mm/s scanning speed; for CD2 film: 100 mg/ml glucosamine hydrochloride, 60% laser power, 150 mm/s scanning speed.

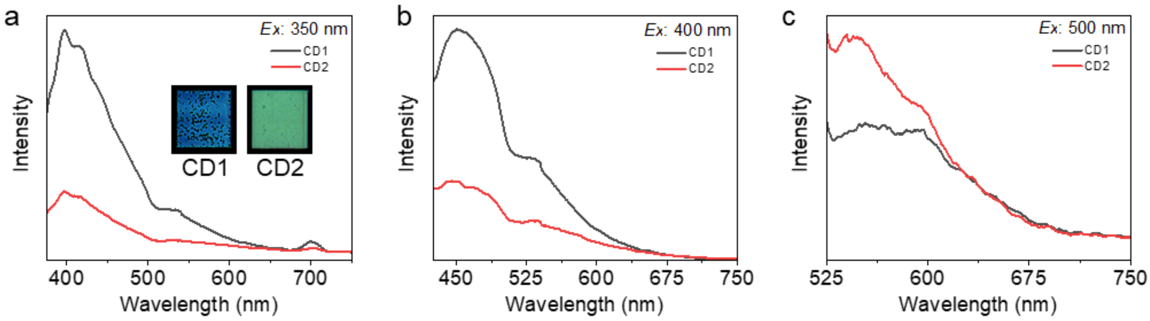


**Supplementary Figure 13. Fluorescence spectra of two carbon dot films under different excitation wavelengths of (a) 350 nm, (b) 400 nm, and (c) 500 nm.** Parameters for the preparation of the CD1 film: 100 mg/ml glucosamine hydrochloride, 40% laser power, 150 mm/s scanning speed; for CD2 film: 100 mg/ml glucosamine hydrochloride, 60% laser power, 150 mm/s scanning speed.

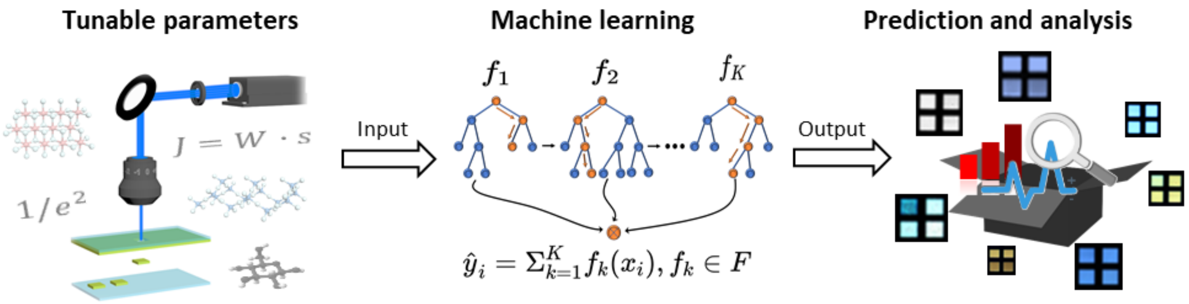


**Supplementary Figure 14. Machine learning was introduced to extend the library from ~2k (experimental) to ~176k (experimental + predicted) datasets.** The parameters involved in the library were: five precursor types, four concentrations of precursors, 15 additives, three laser foci, two donor absorbers, two printing mode, eight laser powers and four scanning speeds.

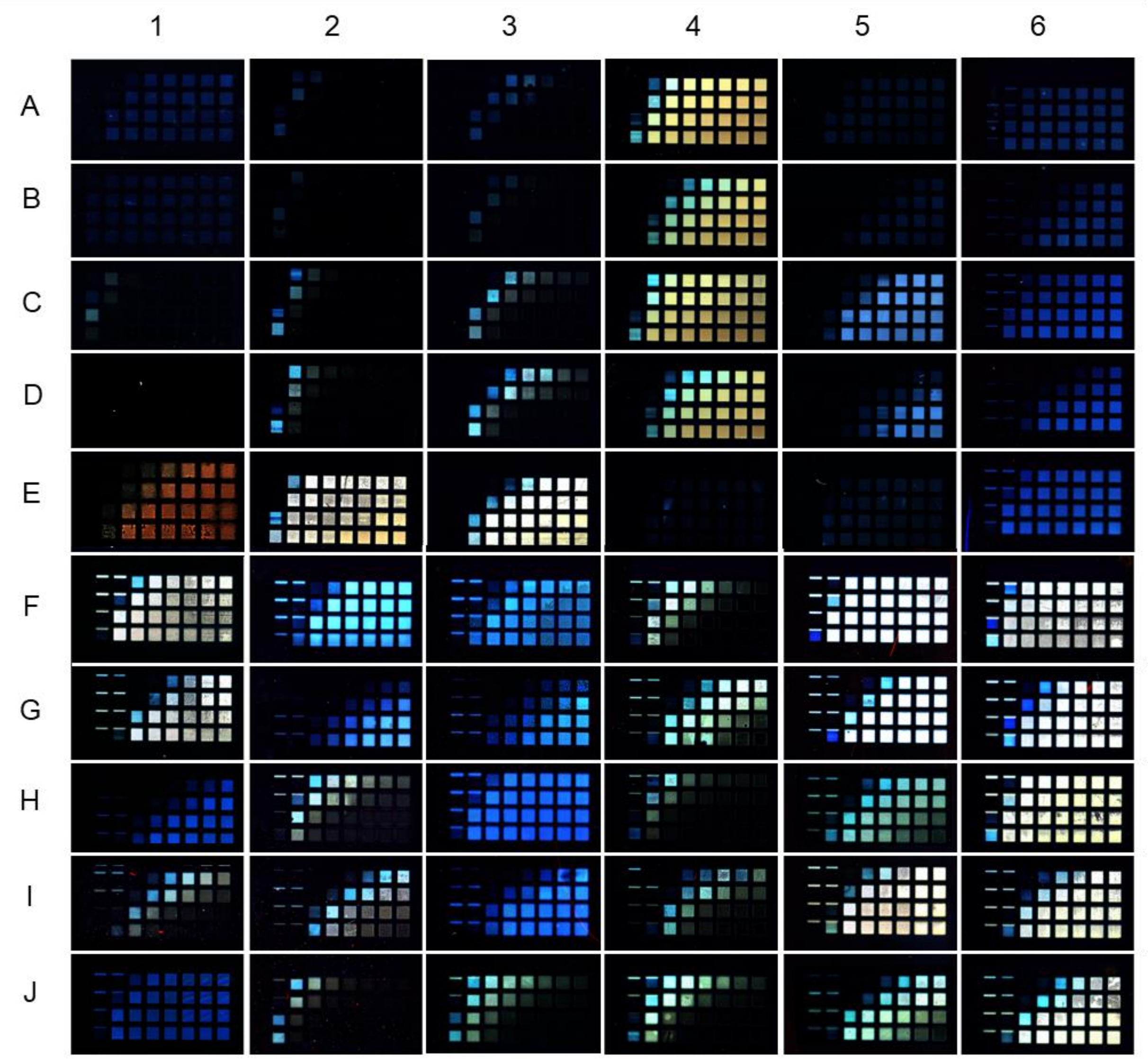


**Supplementary Figure 15. Fluorescent film library achieved by nanoprinting-assisted flash (nanoFlash) synthesis.** 1920 different 1 mm² material films (constant contrast and brightness for red, green, and blue channel) were generated with individual synthesis parameters (see Supplementary Information Table S1).

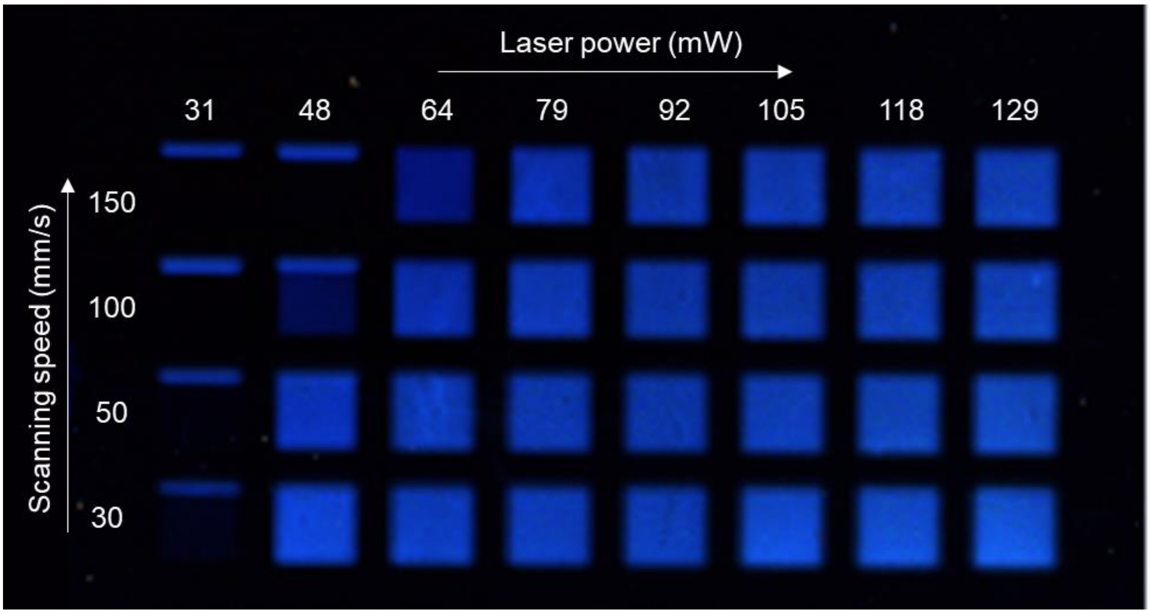


**Supplementary Figure 16.** Different laser powers and scanning speeds in each section of fluorescent film library.

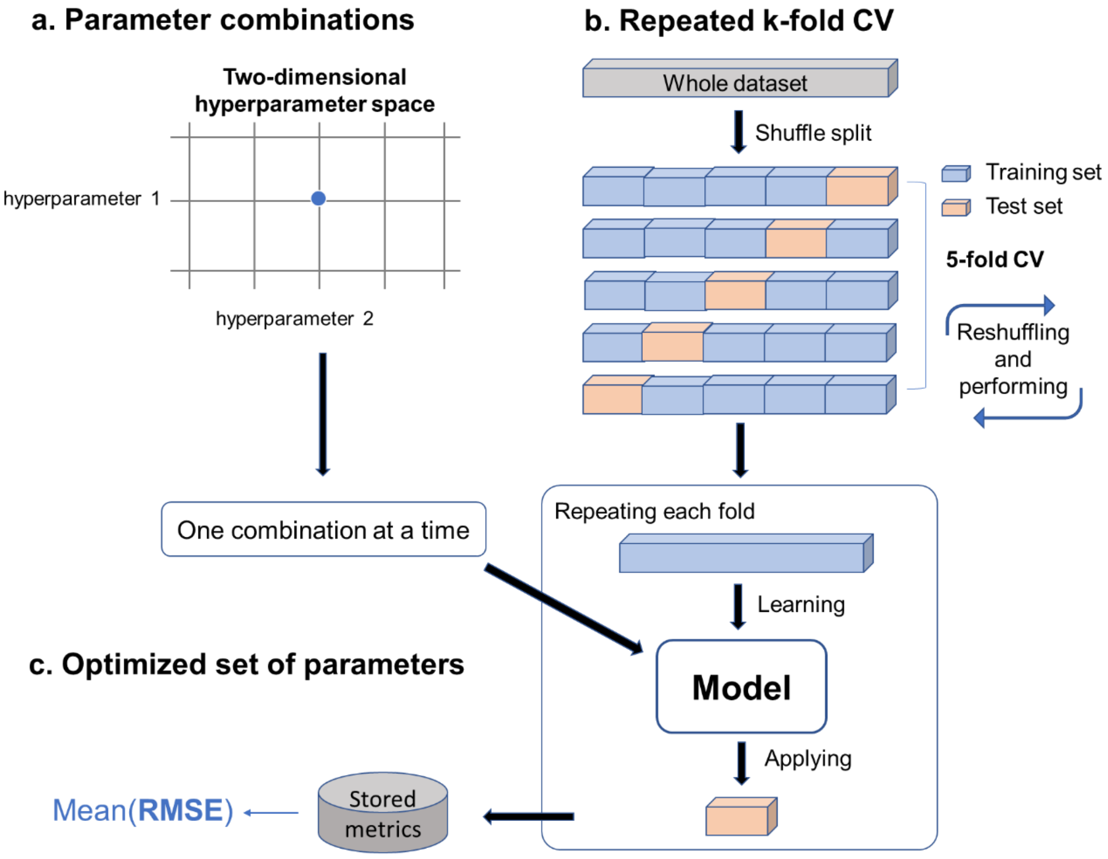


**Supplementary Figure 17. Scheme of the machine learning process.** a) Parameter combinations. The algorithm takes one combination of hyperparameter values in the grid search time. b) Illustration of 5-fold cross-validation (CV) procedure. The whole datasets were randomly separated into training datasets and test datasets at a ratio of 4:1. c) Optimized set of parameters. After taking all the combinations, the algorithm outputs the best hyperparameters.

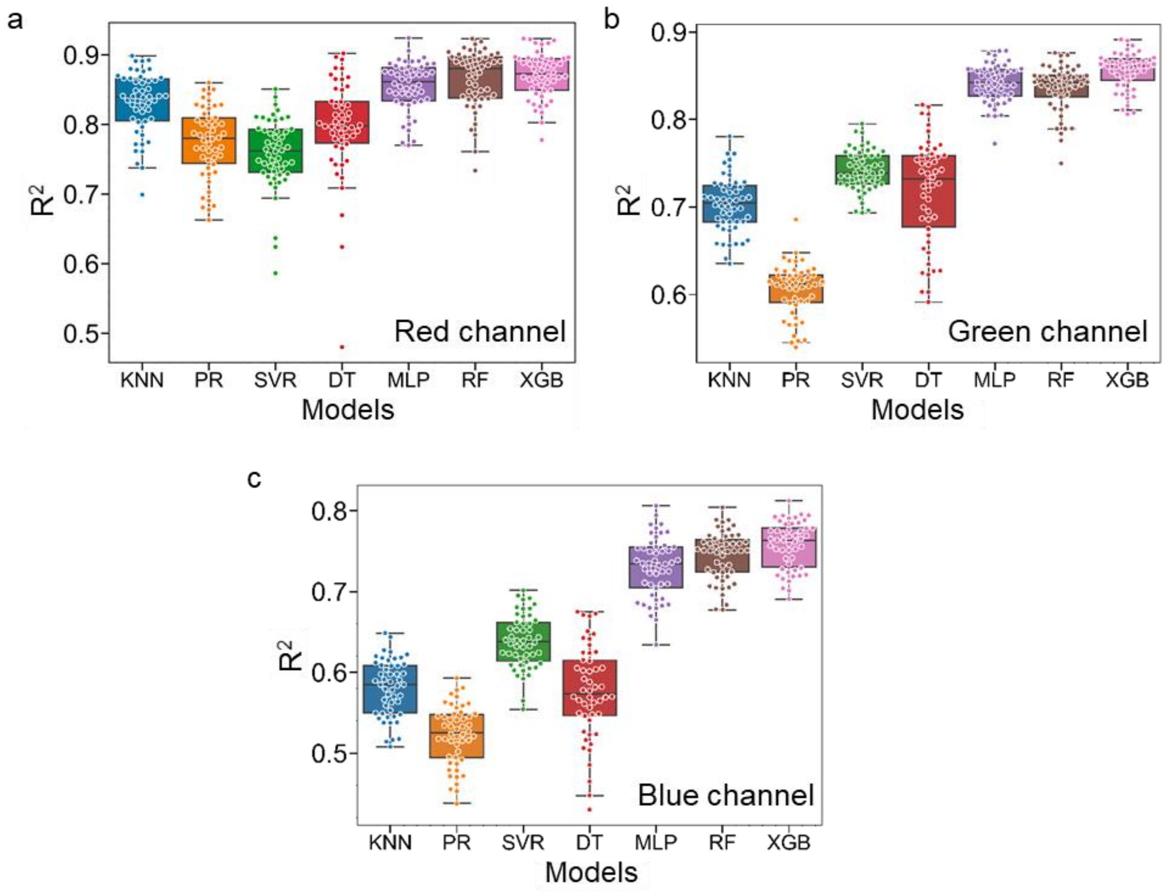


**Supplementary Figure 18.** Determination coefficient ($R^2$) of different models for (a) red channel, (b) green channel and (c) blue channel with n = 50 for each model (see Methods section and Supplementary Figure 17). Box plot: center line, median; box limits, upper and lower quartiles; whiskers, 1.5x interquartile range; points, outliers.

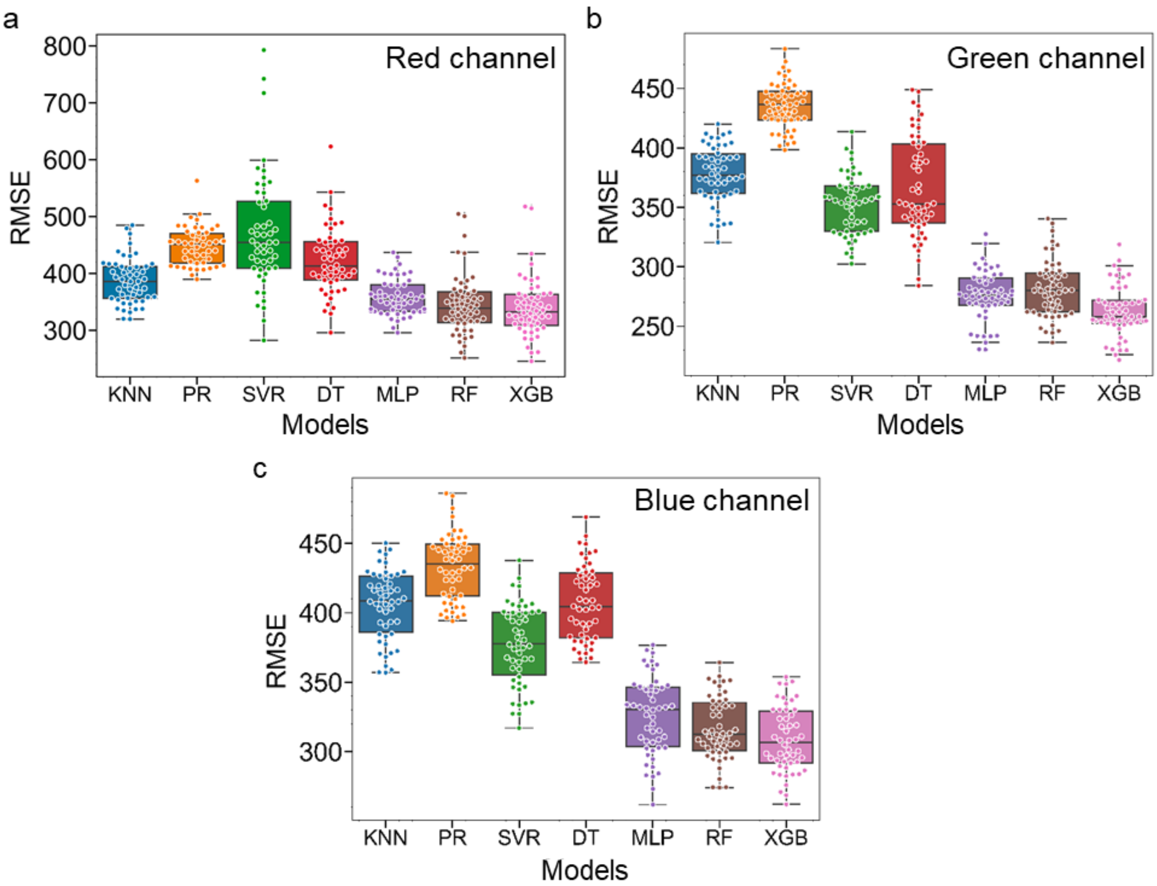


**Supplementary Figure 19.** Root mean squared error (RMSE) of different models for (a) red channel, (b) green channel, (c) blue channel with n = 50 for each model. Box plot: center line, median; box limits, upper and lower quartiles; whiskers, 1.5x interquartile range; points, outliers.

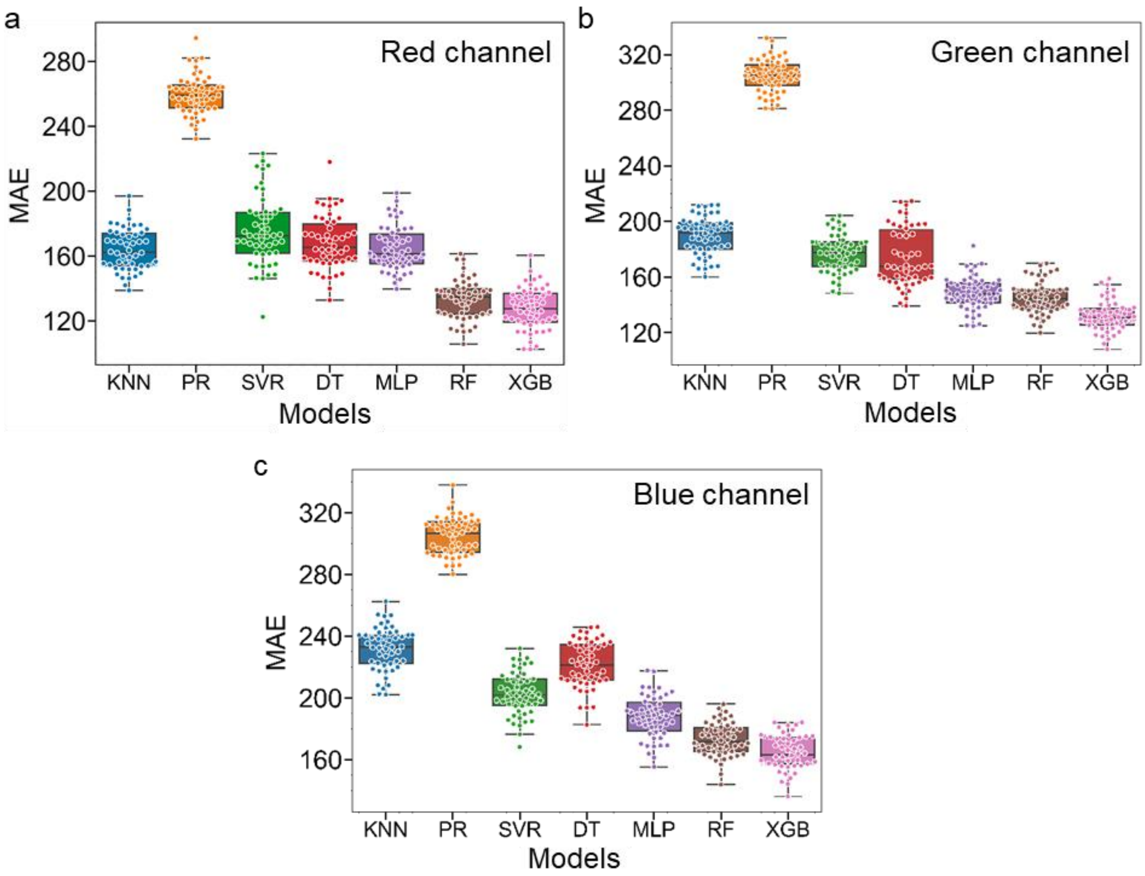


**Supplementary Figure 20.** Mean absolute error (MAE) of different models for (a) red channel, (b) green channel, (c) blue channel with n = 50 for each model. Box plot: center line, median; box limits, upper and lower quartiles; whiskers, 1.5x interquartile range; points, outliers.

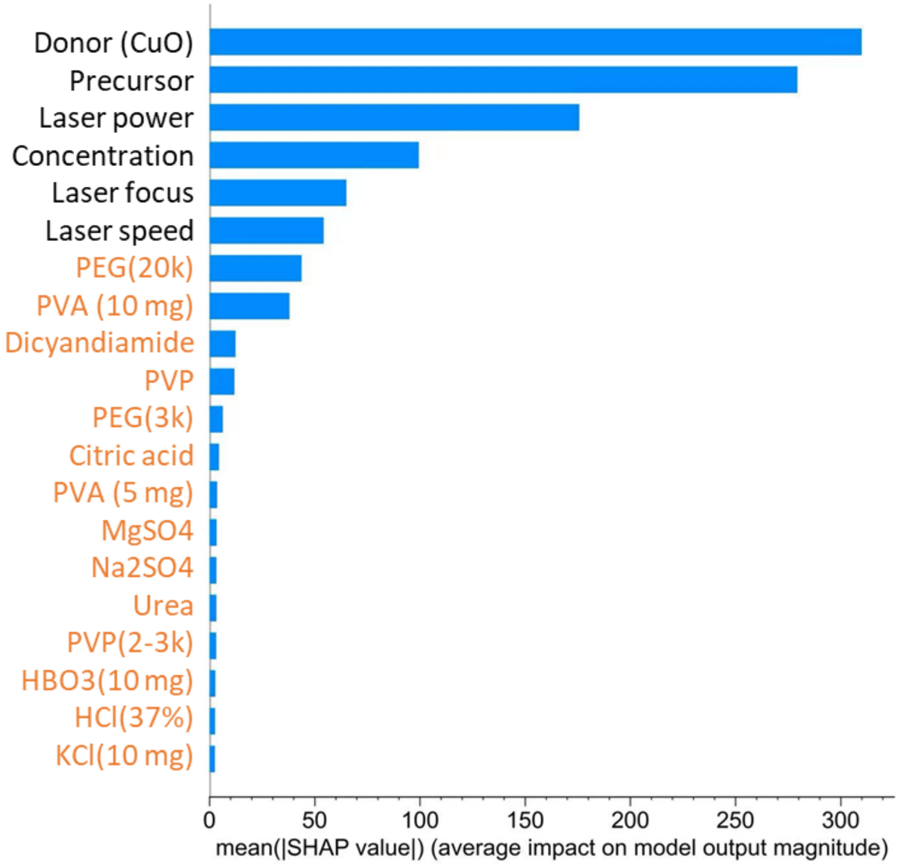


**Supplementary Figure 21.** SHAP feature importance measured as the mean absolute Shapley values for red channel.

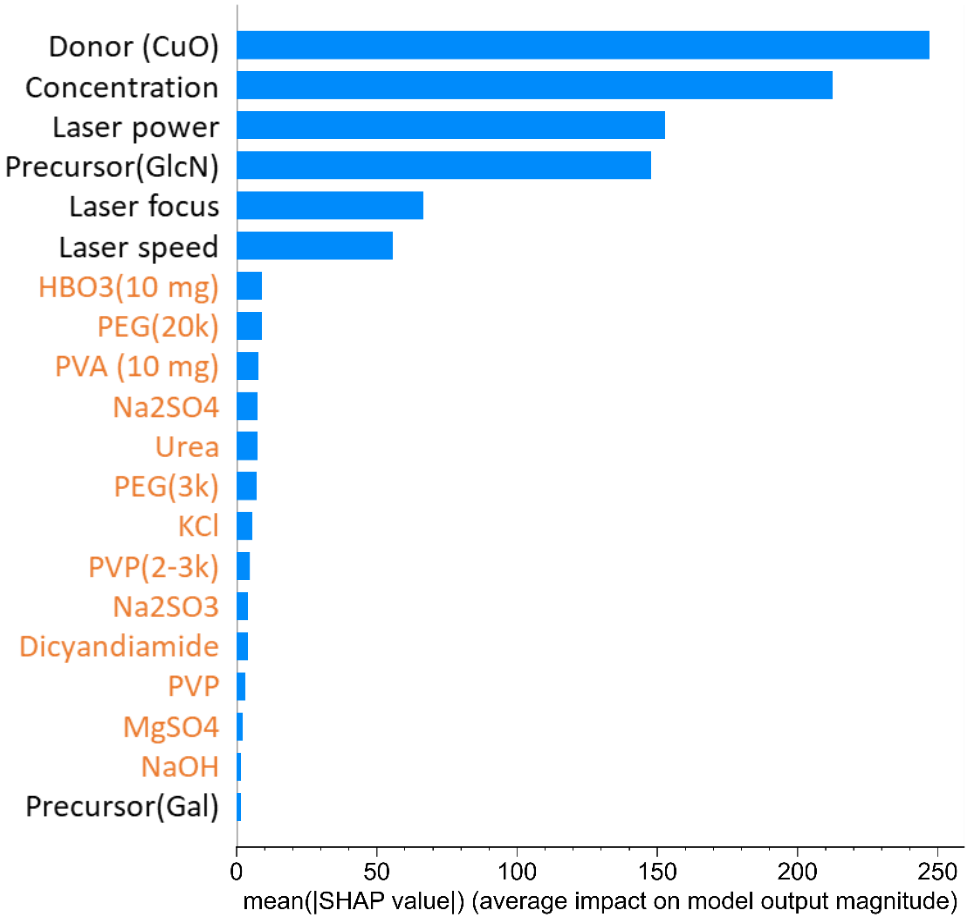


**Supplementary Figure 22.** SHAP feature importance measured as the mean absolute Shapley values for green channel.

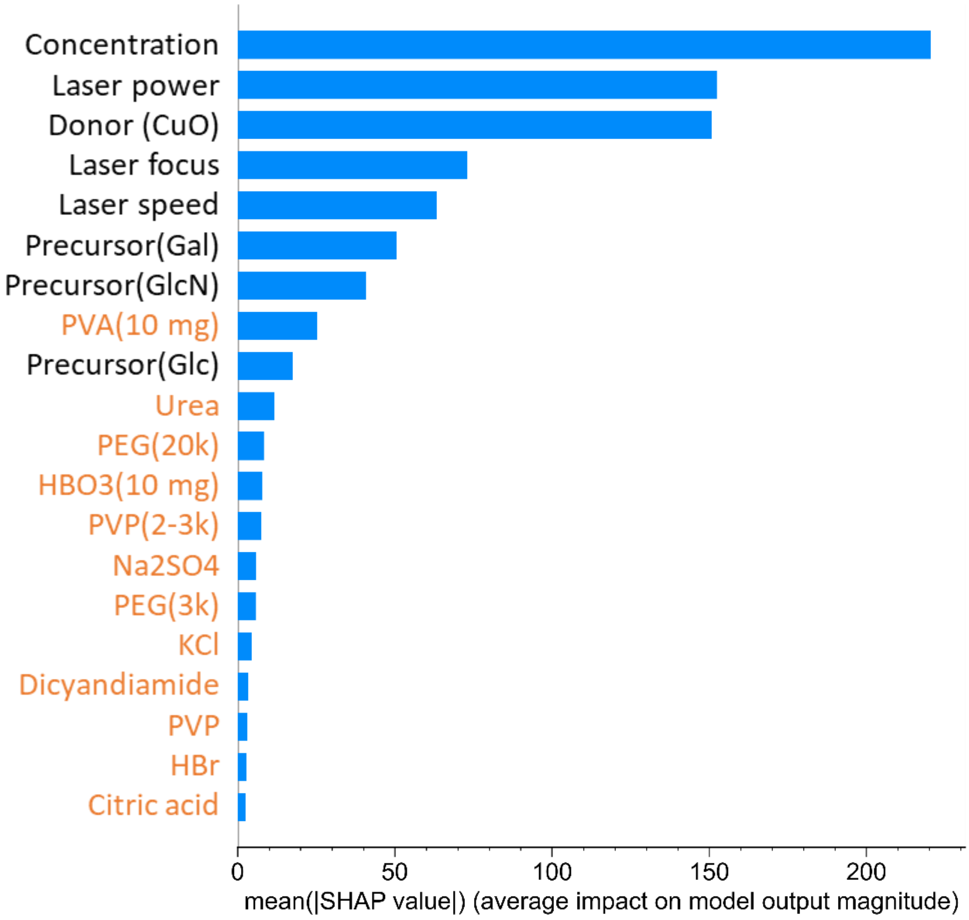


**Supplementary Figure 23.** SHAP feature importance measured as the mean absolute Shapley values for blue channel.

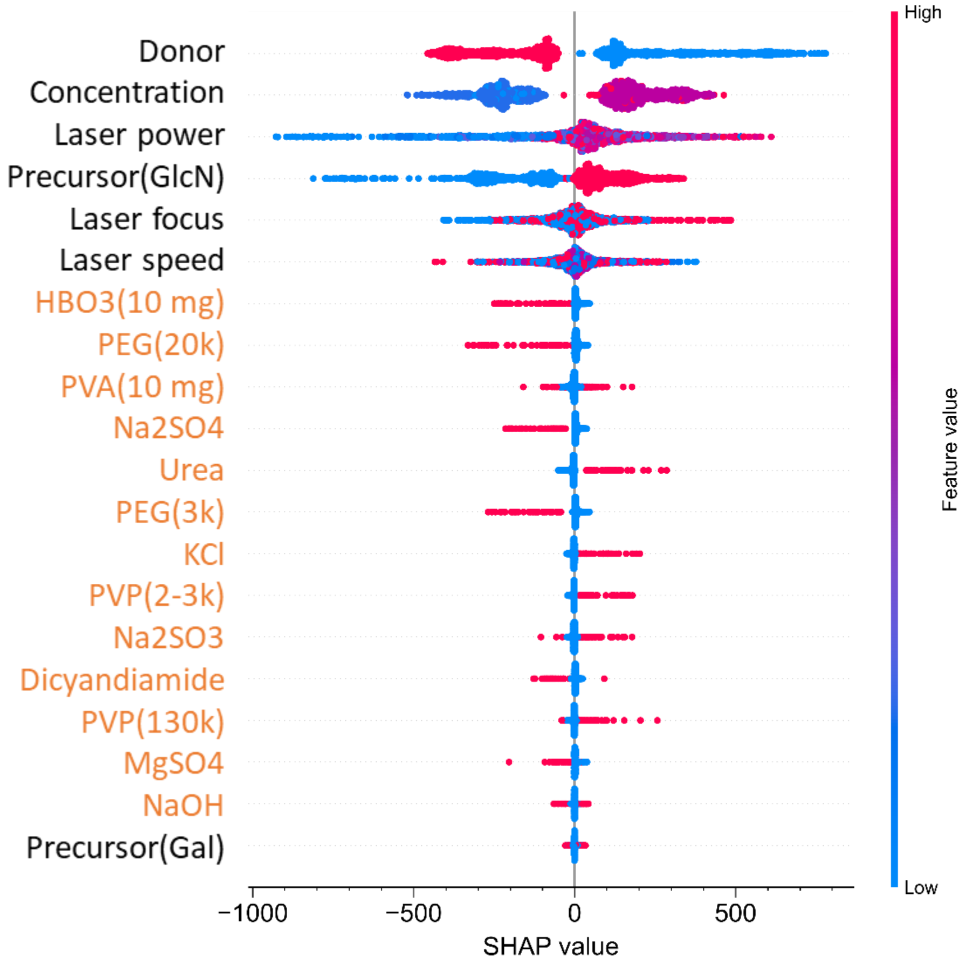


**Supplementary Figure 24.** SHAP summary plot in XGB model for green fluorescence intensity. The color represents the value of the feature from low (blue) to high (red).

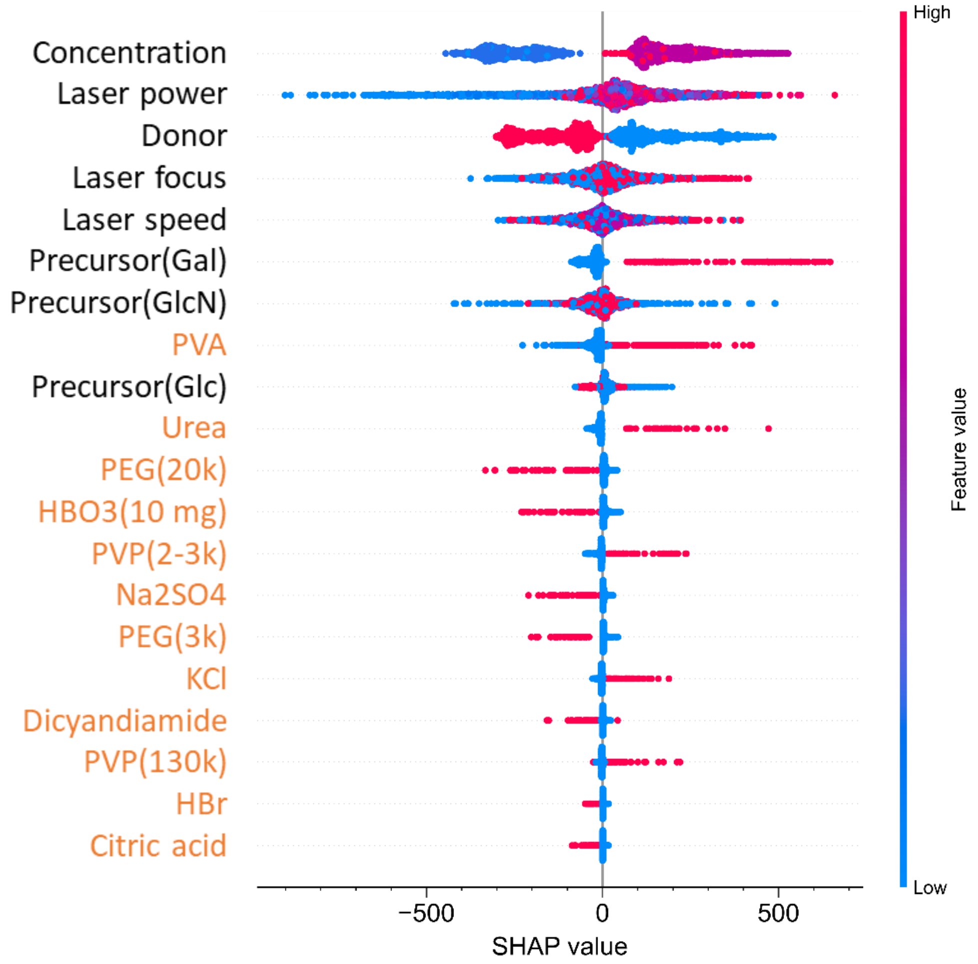


**Supplementary Figure 25.** SHAP summary plot in XGB model for blue fluorescence intensity. The color represents the value of the feature from low (blue) to high (red).

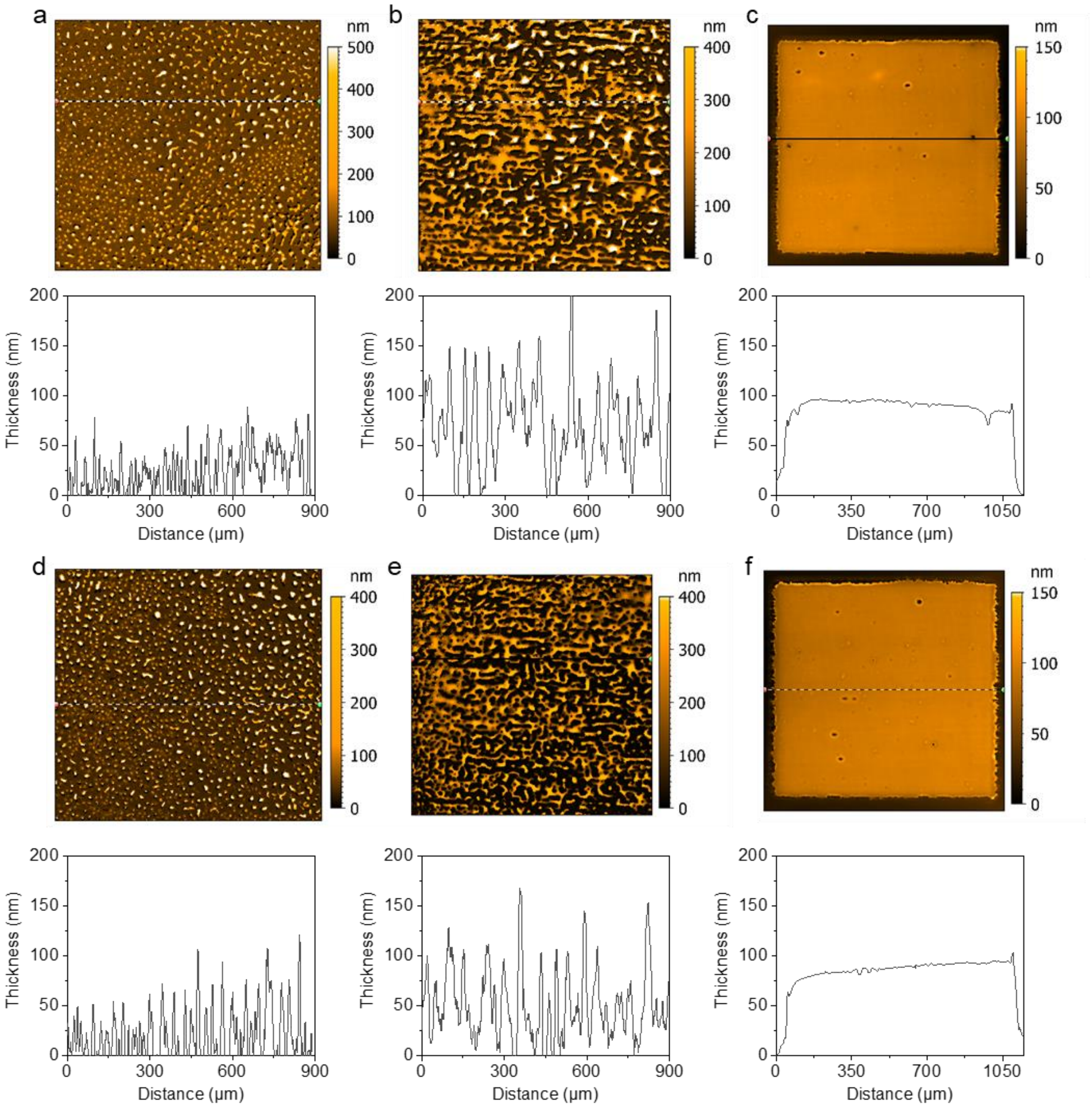


**Supplementary Figure 26.** Height maps and profiles of PUF patterns with (a, d) particle-like droplets (N-acetylglucosamine as precursor, 49 mW, 120 mm/s), (b, e) stripe-like droplets (N-acetylglucosamine as precursor, 49 mW, 130 mm/s), and (c, f) continuous films (galactose as precursor, 98 mW, 100 mm/s).

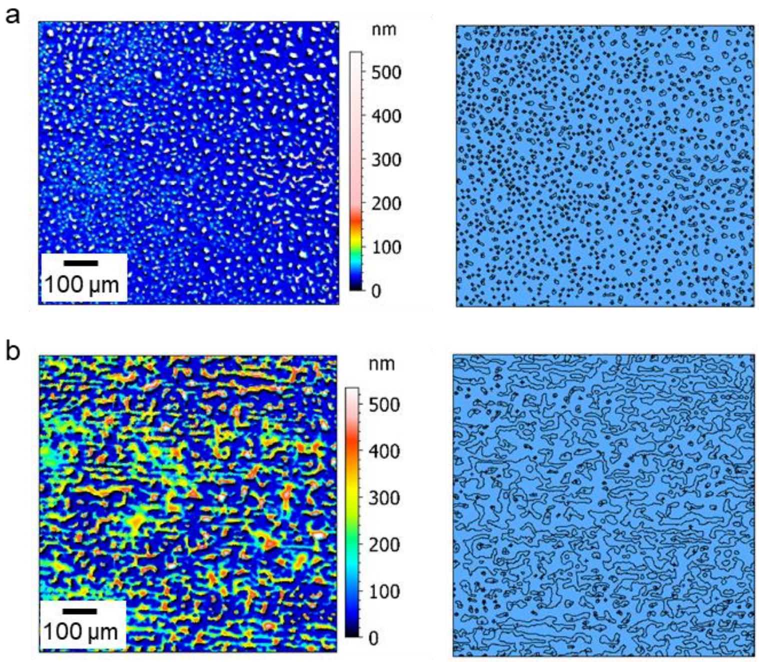


**Supplementary Figure 27.** Two PUF patterns were generated with different laser parameters (a) 49 mW, 120 mm/s, (b) 49 mW, 130 mm/s. Their topography was characterized by WLI and extracted by edge detection.

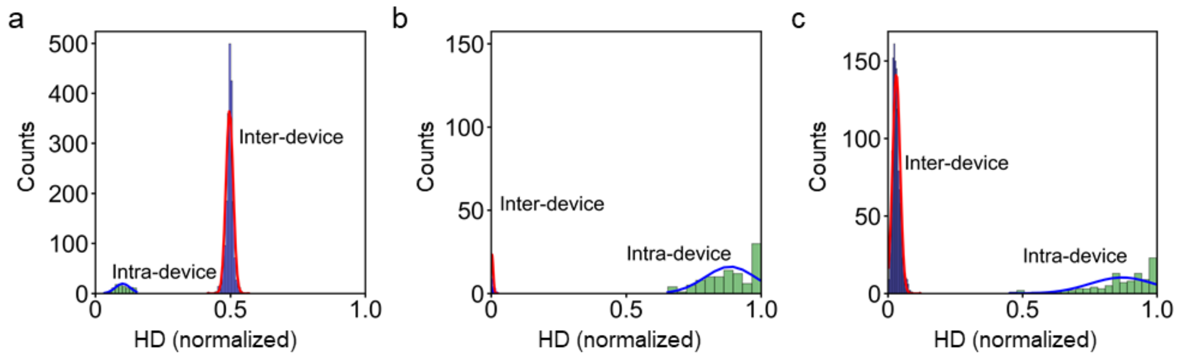


**Supplementary Figure 28.** Device uniqueness of the PUF patterns was characterized by (a) inter-device Hamming distance (HD) for the topography channel, (b) inter-device LoFTR matching for blue fluorescence, (c) inter-device LoFTR matching for the topography channel.

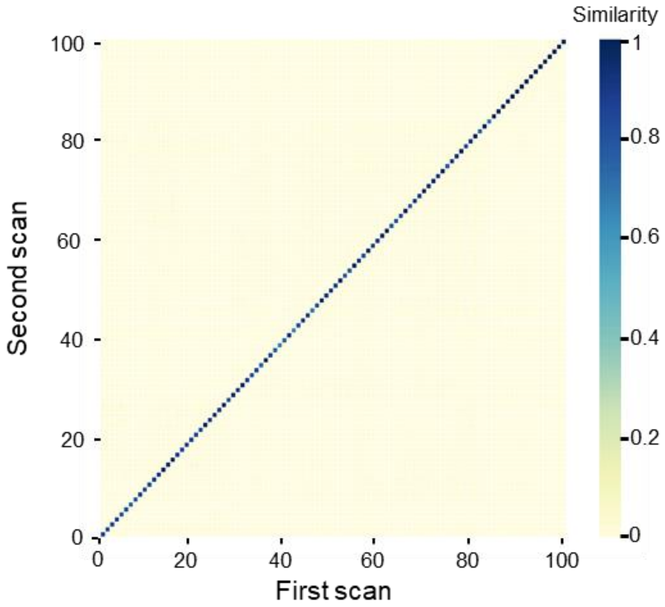


**Supplementary Figure 29.** Heat map of FL similarity values (match ratio, referenced to maximum measured match number: 4256) obtained from 100 unique PUF patterns, created using the same nanoFlash transfer parameters.

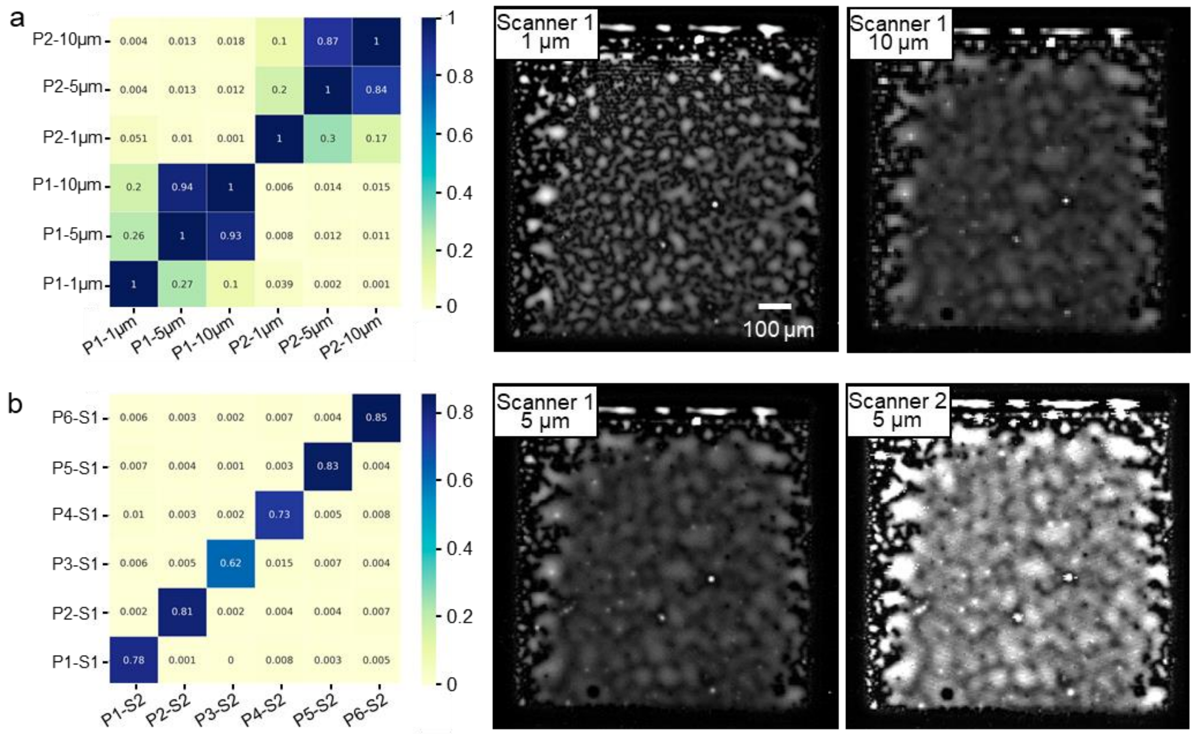


**Supplementary Figure 30. Investigation of the practical fluorescence authentication.** (a) Similarity values between scans of the same PUF pattern with different scanning resolutions. (b) Similarity values between the results from different scanners (Molecular Devices Genepix 4000B, Innopsys Innoscan 1100AL). Fluorescence was scanned in the green channel (Innopsys Innoscan 1100AL Ex: 532 nm, Em: 597.5 – 612.5 nm; Molecular Devices Genepix 4000B Ex: 532 nm, Em: 557.5 – 592.5 nm).

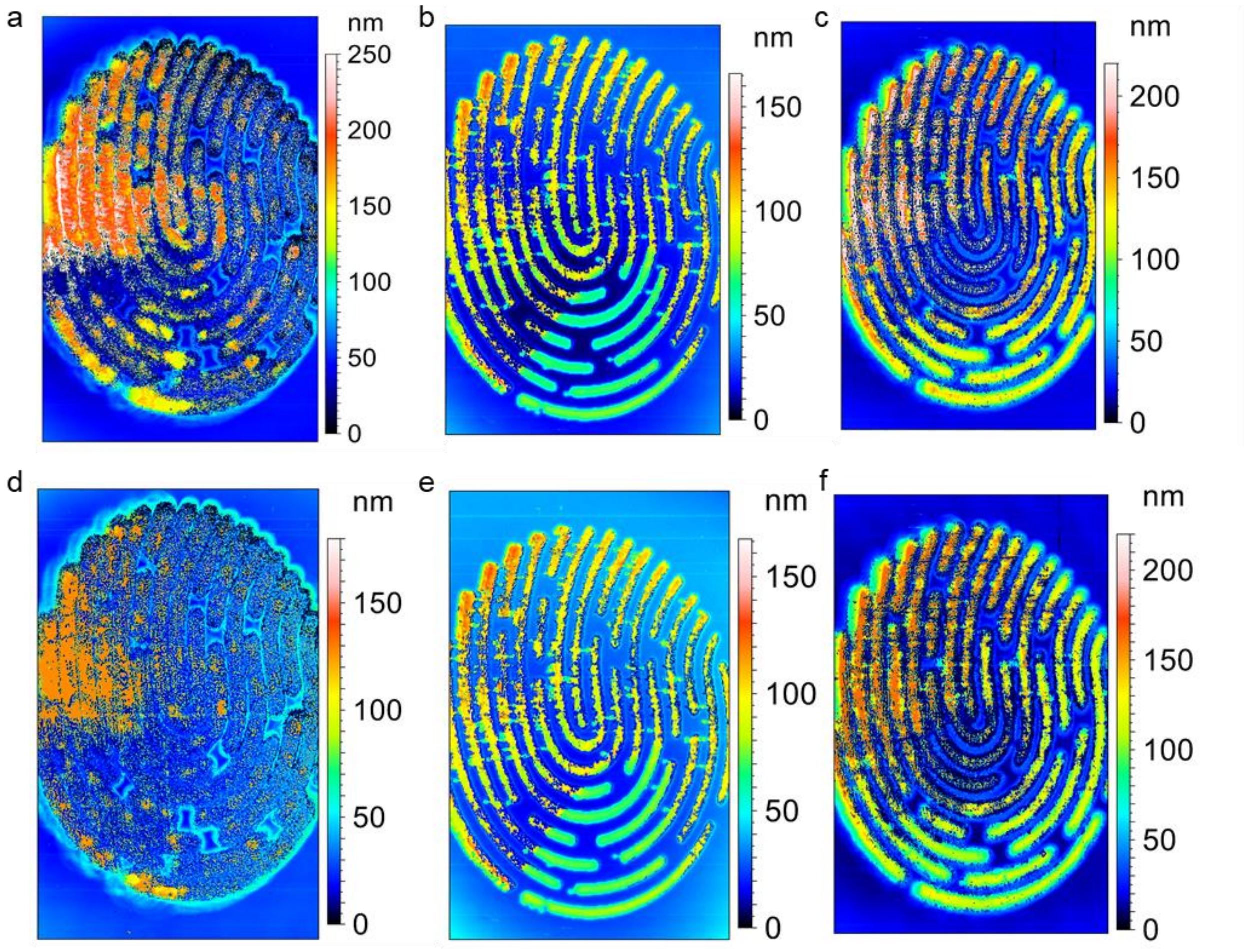


**Supplementary Figure 31.** 2D height map of the first 3 fingerprints in Fig. 5 directly after printing (a-c) and 2 months after printing (d-f).

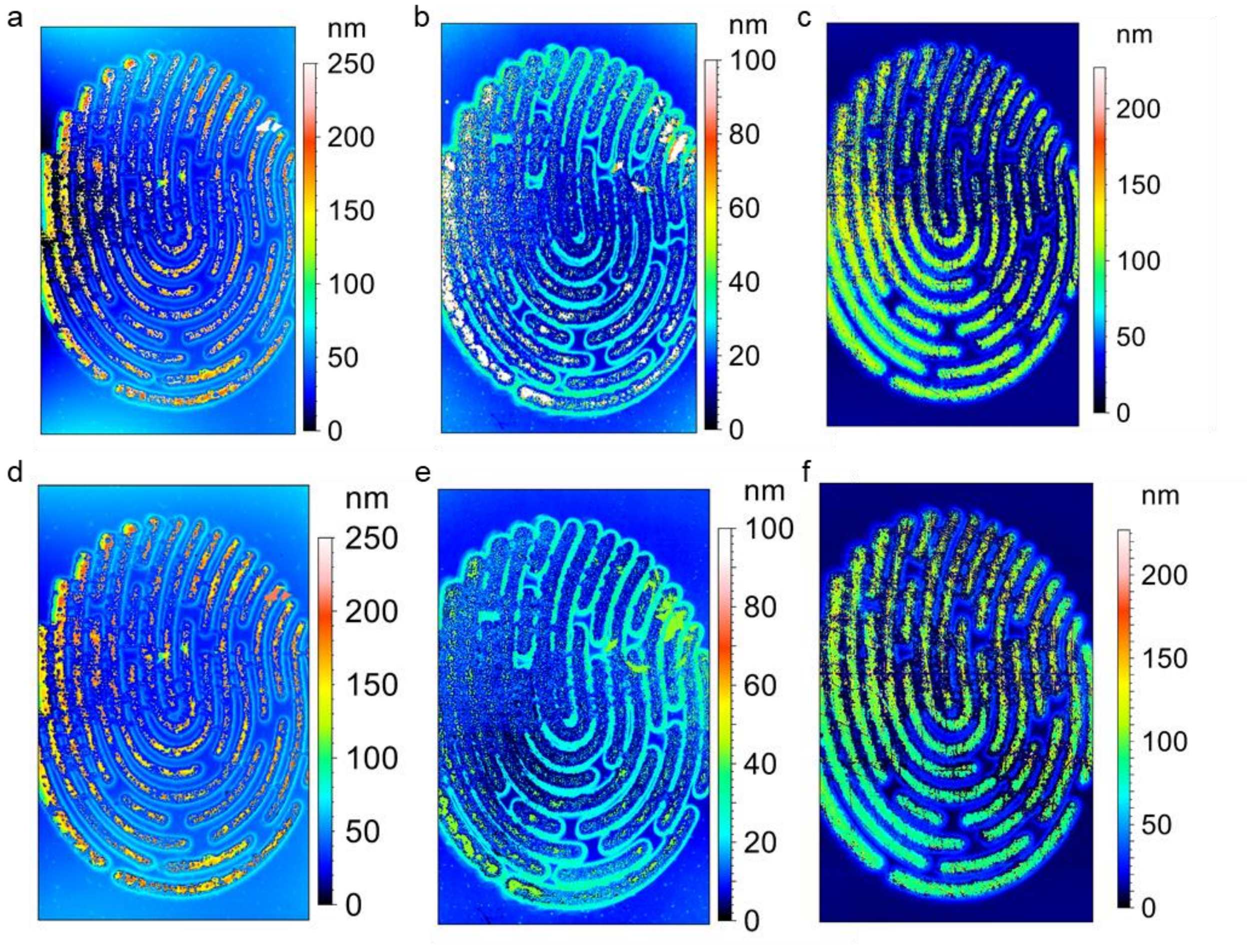


**Supplementary Figure 32.** 2D height map of the last 3 fingerprints in Fig. 5 directly after printing (a-c) and 2 months after printing (d-f).

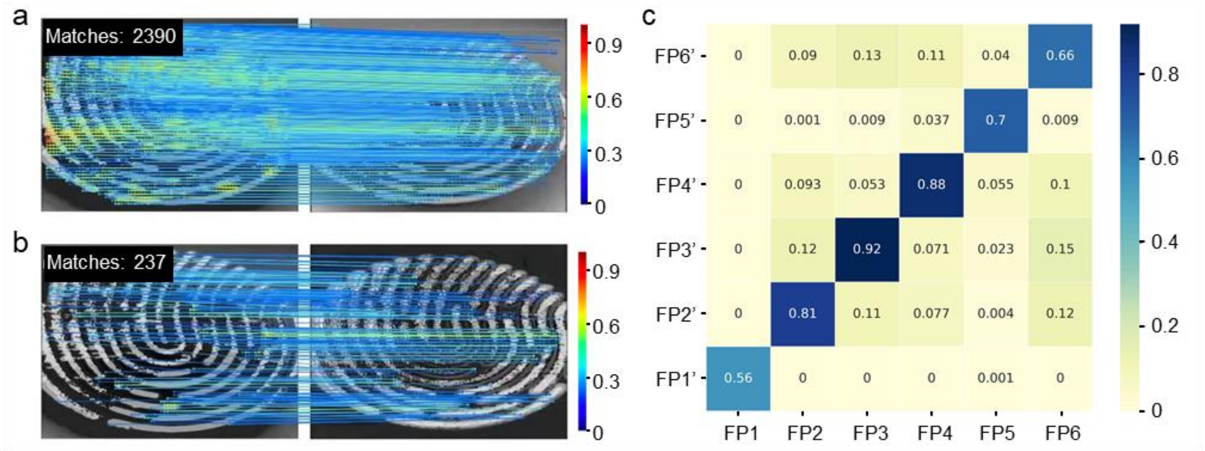


**Supplementary Figure 33. Similarity analysis of topography patterns by LoFTR algorithm.** (a) Intracorrelation for the same PUF pattern of two independent scans. (b) Intercorrelation between two different PUF patterns. (c) Heat map of WLI similarity values (match ratio, referenced to maximum measured match number: 4256) obtained from the fingerprint patterns. FP1–FP6 and FP1'–FP6' are the readout of the fresh patterns and the patterns after 2 months respectively, which had been shown in Fig. S34).

**Supplementary Table 1.** Elemental composition and ratio of the precursor and nanoFlash films.

| Sample | Carbon (at%) | Nitrogen (at%) | Oxygen (at%) | Chlorine (at%) | Substrate signal (Si, Ca…)(at%) | C/N | C/O | C/Cl | N/Cl | O/Cl |
|---|---|---|---|---|---|---|---|---|---|---|
| Precursor film | 49.4 | 7.9 | 36.4 | 6.3 | 0 | 6.2 | 1.4 | 7.8 | 1.3 | 5.8 |
| NanoFlash film | 61.2 | 8.2 | 27.9 | 2.7 | 0.6 | 7.4 | 2.2 | 23.2 | 3.2 | 10.5 |

**Supplementary Table 2.** XPS deconvolution and functional group assignments.

| Element | Binding energy (eV) | Assignments | Precursor film (at%) | NanoFlash film (at%) |
|---|---|---|---|---|
| C 1s | 285 | C-C | 7.2 | 19.5 |
| C 1s | ~ 286.4 | C-O / C-N | 34.2 | 30.2 |
| C 1s | ~ 288.0 | N-C=O / C=O | 7.9 | 9.8 |
| C 1s | ~ 289.3 | COO | - | 1.3 |
| N 1s | ~399.7 | -$NH_2$ / N-C=O from heterocycles | 1.4 | 4.9 |
| N 1s | 401.5 | $^{+}NH_3$ (Cl-) | 6.5 | 3.3 |
| O 1s | 531.2 | N-C=O | - | 1.7 |
| O 1s | ~532.8 | C-O / C=O | 35.6 | 24.6 |
| O 1s | 534-535 | $OH_{ads}$ | 0.5 | 1.0 |
| Cl 2p | ~198 | $Cl^{-}$($^{+}NH_3$) | 6.3 | 2.7 |

**Supplementary Table 3.** Detailed synthesis parameters for the nanofilms in the library.

| No. | Precursor | Amount (mg) | Additive | Laser focus | Donor | Printing type | Thickness (nm) |
|---|---|---|---|---|---|---|---|
| A1 | Glucose | 50 | No | 4.32 | CuO | Bitmap | 700 |
| B1 | Glucose | 50 | No | 4.62 | CuO | Bitmap | 700 |
| C1 | Glucosamine | 50 | No | 4.62 | CuO | Bitmap | 35 |
| D1 | Toluenesulfonic acid | 50 | 10 mg PEG (20 k) | 4.62 | CuO | Bitmap | 120 |
| E1 | Toluenesulfonic acid | 50 | 10 mg PVA (9.5 k) | 4.62 | CuO | Bitmap | 200 |
| F1 | Glucosamine | 150 | No | 4.62 | $Fe_2O_3$ | Filled | 202 |
| G1 | Glucosamine | 150 | No | 4.32 | $Fe_2O_3$ | Filled | 205 |
| H1 | Galactose | 50 | No | 4.32 | $Fe_2O_3$ | Filled | 164 |
| I1 | Glucosamine | 100 | 10 mg $HBO_3$ | 4.32 | CuO | Filled | 190 |
| J1 | Galactose | 50 | No | 4.62 | $Fe_2O_3$ | Filled | 203 |
| A2 | Glucosamine | 50 | 50 µl HCl (37%) | 4.62 | CuO | Bitmap | 62 |
| B2 | Glucosamine | 25 | No | 4.62 | CuO | Bitmap | 179 |
| C2 | Glucosamine | 100 | No | 4.62 | CuO | Bitmap | 121 |
| D2 | Glucosamine | 150 | No | 4.62 | CuO | Bitmap | 62 |
| E2 | Glucosamine | 100 | No | 4.62 | $Fe_2O_3$ | Bitmap | 120 |
| F2 | N-Acetylglucosamine | 100 | 10 mg PVA (9.5 k) | 4.62 | CuO | Filled | 183 |
| G2 | N-Acetylglucosamine | 100 | 10 mg PVA (9.5 k) | 4.32 | CuO | Filled | 191 |
| H2 | Glucosamine | 100 | 10 mg PVA (9.5 k) | 4.62 | CuO | Filled | 34 |
| I2 | Glucosamine | 100 | 10 mg PVA (9.5 k) | 4.32 | CuO | Filled | 32 |
| J2 | Glucosamine | 100 | 10 mg $HBO_3$ | 4.62 | CuO | Filled | 191 |
| A3 | Glucosamine | 50 | 50 µl HCl (37%) | 4.32 | CuO | Bitmap | 149 |
| B3 | Glucosamine | 25 | No | 4.32 | CuO | Bitmap | 99 |
| C3 | Glucosamine | 100 | No | 4.32 | CuO | Bitmap | 126 |
| D3 | Glucosamine | 150 | No | 4.32 | CuO | Bitmap | 579 |
| E3 | Glucosamine | 100 | No | 4.32 | $Fe_2O_3$ | Bitmap | 99 |
| F3 | Galactose | 100 | 10 mg PVA (9.5 k) | 4.62 | CuO | Filled | 167 |
| G3 | Galactose | 100 | 10 mg PVA (9.5 k) | 4.32 | CuO | Filled | 171 |
| H3 | Galactose | 100 | 5 mg PVA (9.5 k) | 4.62 | CuO | Filled | 237 |
| I3 | Galactose | 100 | 5 mg PVA (9.5 k) | 4.32 | CuO | Filled | 187 |

| | | | | | | | |
|---|---|---|---|---|---|---|---|
| J3 | Glucosamine | 100 | 10 mg NaOH | 4.62 | CuO | Filled | 206 |
| A4 | Glucosamine | 50 | No | 4.62 | $Fe_2O_3$ | Bitmap | 105 |
| B4 | Glucosamine | 50 | No | 4.32 | $Fe_2O_3$ | Bitmap | 65 |
| C4 | Glucosamine | 50 | 50 µl HCl (37%) | 4.62 | $Fe_2O_3$ | Bitmap | 50 |
| D4 | Glucosamine | 50 | 50 µl HCl (37%) | 4.32 | $Fe_2O_3$ | Bitmap | 50 |
| E4 | Glucose | 50 | 50 µl HCl (37%) | 4.62 | $Fe_2O_3$ | Bitmap | 179 |
| F4 | Glucosamine | 100 | 10 mg KCl | 4.62 | CuO | Filled | 190 |
| G4 | Glucosamine | 100 | 10 mg KCl | 4.32 | CuO | Filled | 190 |
| H4 | Glucosamine | 100 | 10 mg $Na_2SO_4$ | 4.62 | CuO | Filled | 186 |
| I4 | Glucosamine | 100 | 10 mg $Na_2SO_4$ | 4.32 | CuO | Filled | 174 |
| J4 | Glucosamine | 100 | 10 mg $Na_2SO_3$ | 4.62 | CuO | Filled | 186 |
| A5 | Glucose | 50 | 5 µl HCl (37%) | 4.62 | $Fe_2O_3$ | Bitmap | 75 |
| B5 | Glucose | 50 | 5 µl HCl (37%) | 4.32 | $Fe_2O_3$ | Bitmap | 134 |
| C5 | Glucose | 50 | 10 mg PVP (2.5 k) | 4.62 | $Fe_2O_3$ | Bitmap | 121 |
| D5 | Glucose | 50 | 10 mg PVP (2.5 k) | 4.32 | $Fe_2O_3$ | Bitmap | 146 |
| E5 | Glucose | 50 | 10 mg PEG (3 k) | 4.62 | $Fe_2O_3$ | Bitmap | 122 |
| F5 | Glucose+ Glucosamine | 50+50 | No | 4.62 | $Fe_2O_3$ | Filled | 162 |
| G5 | Glucose+ Glucosamine | 50+50 | No | 4.32 | $Fe_2O_3$ | Filled | 155 |
| H5 | Glucosamine | 100 | 10 mg NaOH | 4.32 | CuO | Filled | 167 |
| I5 | Glucosamine | 100 | 10 mg Citric acid | 4.32 | $Fe_2O_3$ | Filled | 193 |
| J5 | Glucosamine | 100 | 10 mg $Na_2SO_3$ | 4.32 | CuO | Filled | 182 |
| A6 | N-Acetylglucosamine | 25 | No | 4.62 | $Fe_2O_3$ | Bitmap | 210 |
| B6 | N-Acetylglucosamine | 25 | No | 4.32 | $Fe_2O_3$ | Bitmap | 198 |
| C6 | N-Acetylglucosamine | 50 | No | 4.62 | $Fe_2O_3$ | Bitmap | 94 |
| D6 | N-Acetylglucosamine | 50 | No | 4.32 | $Fe_2O_3$ | Bitmap | 192 |
| E6 | N-Acetylglucosamine | 100 | No | 4.62 | $Fe_2O_3$ | Bitmap | 195 |
| F6 | Glucosamine | 100 | 10 mg Urea | 4.62 | $Fe_2O_3$ | Filled | 168 |
| G6 | Glucosamine | 100 | 10 mg Urea | 4.32 | $Fe_2O_3$ | Filled | 199 |
| H6 | Glucosamine | 100 | 10 mg Dicyandiamide | 4.62 | $Fe_2O_3$ | Filled | 171 |
| I6 | Glucosamine | 100 | 10 mg Dicyandiamide | 4.32 | $Fe_2O_3$ | Filled | 194 |
| J6 | Glucosamine | 100 | 10 mg Guanidine | 4.32 | $Fe_2O_3$ | Filled | 195 |

**Supplementary Table 4.** Reported applications of fluorescent films and the potential use of our nanoFlash approach and nanofilm library for each specific field.

| Application | Reported approaches | | | | Our approach | |
|---|---|---|---|---|---|---|
| | **Materials** | **Strategy** | **Properties** | **Citation** | **Advantages** | **Challenges** |
| Color conversion layers | Organic nanoparticles | Surfactant processing | Full spectral gamut with high color purity | *Nat Commun **13**, 1801 (2022)* | 1. Easier to synthesize and more stable than organic fluorophores<br>2. Non-toxic and widely available compared with inorganic quantum dots | Carbon dots generally have a broad FL spectrum, so efforts are required for color purity |
| | CdSe quantum dots | Hybridized with cyanostilbene unit | Photo-tunable dual fluorescent characteristic | *Adv. Mater.* **24**, 4020–4024 (2012) | | |
| | Organic small molecules | BODIPY-containing structure | White-light emission under low-current operation | *Adv. Mater. **26**, 7290–7294 (2014)* | | |
| | CdSe/(CdS/)ZnS/ CdSZnS quantum dots | Multiply passivated green- and red-light-emitting quantum dots | High color reproducibility and full color gamut | Adv. Mater. 22, 3076–3080 (2010) | | |
| | Organic small molecules | Aggregation-induced emission luminogens | Full-color emission and high data transmission rates | ACS Appl. Mater. Interfaces 10, 34418–34426 (2018) | | |
| | Perovskite nanocrystals | Polymer-free films | Tunable color and ultrahigh stability | Chem. Mater. 28, 2902–2906 (2016) | | |
| (O)LED | Fluorescent dye | Reverse intersystem crossing | 1000 cd $m^{-2}$, near 100% internal quantum efficiency | *Adv. Mater.* **26**, 5684–5688 (2014) | High resistance to photobleaching | Brightness of the CD-LED was lower than that of QD-LEDs, which probably can be solved by using hosts like poly(*N*-vinylcarbazole) |
| | Carbon dots | Host–guest doping | 455.2 cd $m^{-2}$, 41% quantum efficiency | *Nanoscale*, **10**, 11211-11221, (2018) | | |
| | Semiconductor quantum dots | Two-step sputtering process | 530 cd $m^{-2}$ | *Adv. Funct. Mater.*, 26: 3454-3461, (2016) | | |
| Bio- & chemosensing | Nanoporous pigments | Cross-responsive array | Unique molecular fingerprints for 19 different toxic chemicals | *Nat. Chem.* **1**, 562–567 (2009) | As a nano-printing technology, our nanoFlash approach allows for the generation of array-based sensors with simultaneous detection and identification of analytes of similar structures. | The design of cross-reaction sensing elements might be challenging for carbon dots. Flexibly tunable surface structures need to be investigated. |
| | Conjugated polydiacetylene polymer | Introducing protective layer | Sequence selective colorimetric sensing | *Nat. Commun.* 4, 3461 (2013) | | |
| | Conjugated polymer | Fluorescence turn-on mechanism | Sensitivities in the micromolar range in solution | *Angew. Chem. Int. Ed.* 49, 8872–8875 (2010) | | |
| | Naphthalene fluorophore | Acyclic cucurbiturils with flexible cavity | High fidelity and low error rate | *J. Am. Chem. Soc.* 139, 14954–14960 (2017) | | |
| | CdSe/ZnS quantum dots | Layer-by-layer deposition | Real-time monitoring of trypsin activity | *J. Am. Chem. Soc.*, 132, 5, 1460–1461, (2010) | | |

**Supplementary Table 5.** Inter- and intracorrelation of films measured with WLI and fluorescence (Fig. 4e and Fig. S28).

| Measurement | Correlation | Mean | Max. | Min. |
|---|---|---|---|---|
| WLI | Intra | 0.870 ± 0.119 | 0.998 | 0.452 |
| | Inter | 0.038 ± 0.018 | 0.120 | 0.001 |
| Fluorescence | Intra | 0.885 ± 0.093 | 1.000 | 0.654 |
| | Inter | 0.003 ± 0.004 | 0.034 | 0.000 |

**Supplementary Table 6.** Detailed synthesis parameters for the artificial fingerprints.

| No. | Precursor | Amount (mg) | Additive | Laser focus | Donor | Printing type | Laser power | Laser speed | Thickness (nm) |
|---|---|---|---|---|---|---|---|---|---|
| 1 | Galactose | 100 | 10 mg PVA (9.5 k) | 4.62 | CuO | Filled | 64 mW | 150 | 167 |
| 2 | Glucosamine | 100 | 10 mg KCl | 4.32 | CuO | Filled | 92 mW | 150 | 190 |
| 3 | Glucosamine | 100 | 10 mg PVP (130 k) | 4.32 | CuO | Filled | 92 mW | 150 | 186 |
| 4 | Glucosamine | 100 | 10 mg Citric acid | 4.32 | $Fe_2O_3$ | Filled | 105 mW | 50 | 193 |
| 5 | Glucosamine | 150 | 10 mg PEG (3 k) | 4.62 | $Fe_2O_3$ | Filled | 79 mW | 50 | 216 |
| 6 | Glucosamine | 150 | 10 mg PEG (3 k) | 4.62 | $Fe_2O_3$ | Filled | 92 mW | 50 | 216 |

**Supplementary Movie 1.** Real-time transfer of a PUF pattern with magnification of the printed pattern.

## References


[1] J. Zhang, Y. Liu, S. Ronneberger, N. V. Tarakina, N. Merbouh, F. F. Loeffler, *Advanced Materials* **2022**, *34*, 2108493.

[2] J. Zhang, Y. Zou, S. Eickelmann, C. Njel, T. Heil, S. Ronneberger, V. Strauss, P. H. Seeberger, A. Savateev, F. F. Loeffler, *Nature Communications* **2021**, *12*, 3224.
