# Supplementary figures and images for: An all-in-one nanoprinting approach for the synthesis of a nanofilm library for unclonable anti-counterfeiting applications
Source: Nat Nanotechnol. 2023 Jun 5;18(9):1027–35. doi: 10.1038/s41565-023-01405-3 (PMC10501905; doi:10.1038/s41565-023-01405-3)

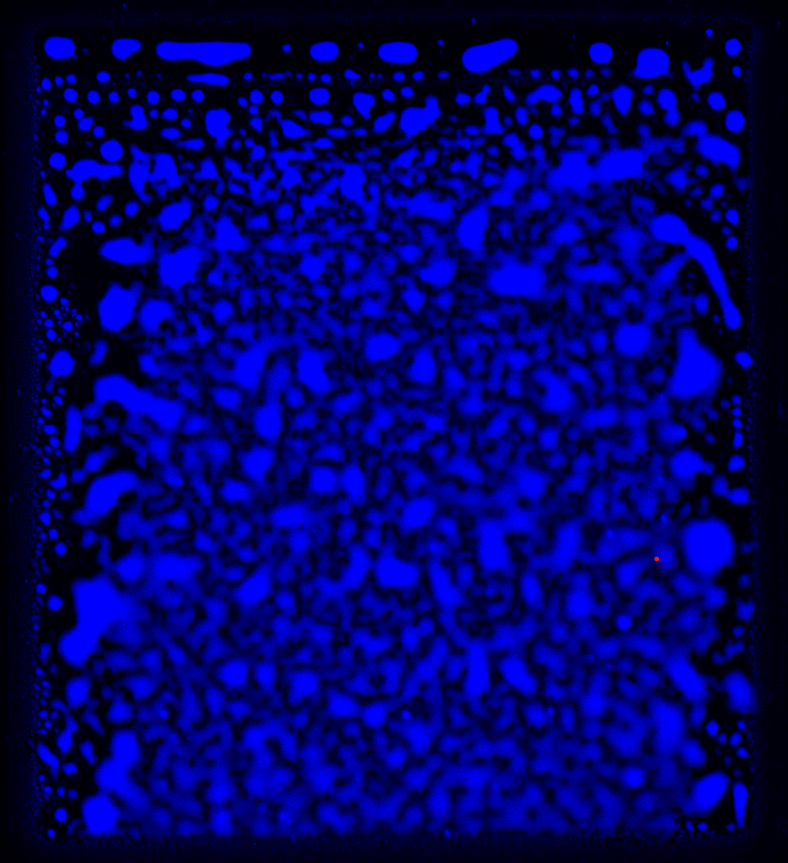

Supplement: Source Data Fig. 2 — Contains: Figure 2bcd source data.xlsx. Figure 2e_blue source data.png. Figure 2e_green source data.png. Figure 2e_red source data.png. (b, c, d) Original data for XPS plots, (e) three fluorescence channel images for correlation analysis. [file 41565_2023_1405_MOESM4_ESM.zip › Figure 2e_blue source data.png]

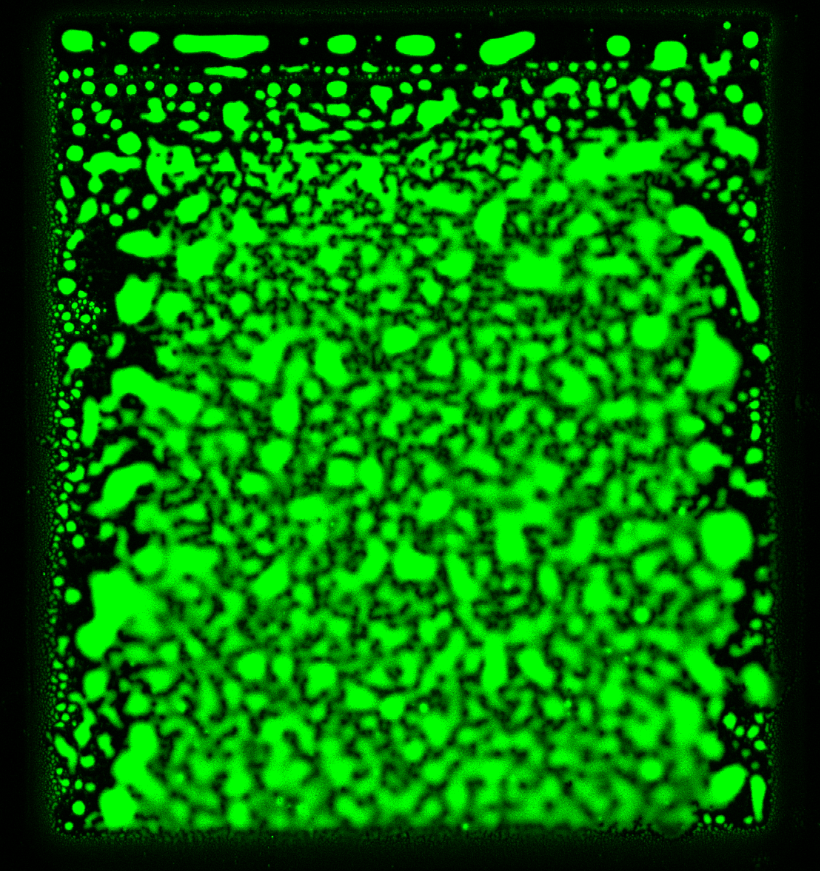

Supplement: Source Data Fig. 2 — Contains: Figure 2bcd source data.xlsx. Figure 2e_blue source data.png. Figure 2e_green source data.png. Figure 2e_red source data.png. (b, c, d) Original data for XPS plots, (e) three fluorescence channel images for correlation analysis. [file 41565_2023_1405_MOESM4_ESM.zip › Figure 2e_green source data.png]

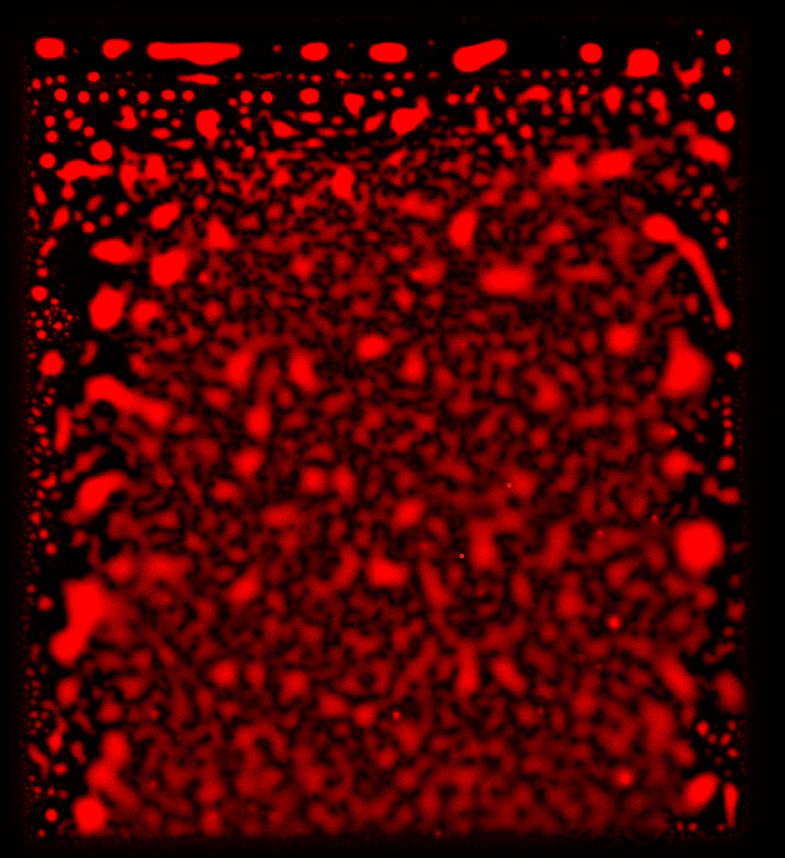

Supplement: Source Data Fig. 2 — Contains: Figure 2bcd source data.xlsx. Figure 2e_blue source data.png. Figure 2e_green source data.png. Figure 2e_red source data.png. (b, c, d) Original data for XPS plots, (e) three fluorescence channel images for correlation analysis. [file 41565_2023_1405_MOESM4_ESM.zip › Figure 2e_red source data.png]
